# Novel Series of Dual NRF2 Inducers and Selective MAO-B Inhibitors for the Treatment of Parkinson’s Disease

**DOI:** 10.3390/antiox11020247

**Published:** 2022-01-27

**Authors:** Pablo Duarte, Patrycja Michalska, Enrique Crisman, Antonio Cuadrado, Rafael León

**Affiliations:** 1 Instituto de Química Médica, Consejo Superior de Investigaciones Científicas (IQM-CSIC), 28006 Madrid, Spain; pablo.duarte@uam.es (P.D.); ecrisman@outlook.com (E.C.); 2 Instituto Teófilo Hernando y Departamento de Farmacología y Terapéutica, Facultad de Medicina, Universidad Autónoma de Madrid, 28029 Madrid, Spain; 3 Chemistry Department, Imperial College London, London W12 0BZ, UK; p.dziama@imperial.ac.uk; 4 Instituto de Investigación Sanitaria La Princesa (IIS-IS), Hospital Universitario de la Princesa, 28006 Madrid, Spain; 5 Departamento de Bioquímica, Facultad de Medicina, Centro de Investigación Biomédica en Red Sobre Enfermedades Neurodegenerativas (CIBERNED), Instituto de Investigación Sanitaria La Paz (IdiPaz), Instituto de Investigaciones Biomédicas ‘Alberto Sols’ UAM-CSIC, Universidad Autónoma de Madrid, 28029 Madrid, Spain; antonio.cuadrado@uam.es

**Keywords:** NRF2, MAO-B, Parkinson’s disease, oxidative stress, neuroinflammation, multitarget

## Abstract

Parkinson’s disease (PD) is the second most prevalent neurodegenerative disease. It is characterized by a complex network of physiopathological events where oxidative stress plays a central role among other factors such as neuroinflammation and protein homeostasis. Nuclear factor-erythroid 2 p45-related factor 2 (NRF2) has a multitarget profile itself as it controls a plethora of cellular processes involved in the progression of the disease. In this line, we designed a novel family of 2-(1*H*-indol-3-yl)ethan-1-amine derivatives as NRF2 inducers with complementary activities. Novel compounds are based on melatonin scaffold and include, among other properties, selective monoamine oxidase B (MAO-B) inhibition activity. Novel multitarget compounds exhibited NRF2 induction activity and MAO-B selective inhibition, combined with anti-inflammatory, antioxidant, and blood–brain barrier permeation properties. Furthermore, they exert neuroprotective properties against oxidative stress toxicity in PD-related in vitro. Hit compound **14** reduced oxidative stress markers and exerted neuroprotection in rat striatal slices exposed to 6-hydroxydopamine or rotenone. In conclusion, we developed a promising family of dual NRF2 inducers and selective MAO-B inhibitors that could serve as a novel therapeutic strategy for PD treatment.

## 1. Introduction

Parkinson’s disease (PD) is the second most prevalent neurodegenerative disease, affecting 2–3% population aged 65 years or over [1]. PD is characterized by a progressive loss of dopaminergic neurons in the substantia nigra pars compacta (SNpc), dopamine levels reduction and the presence of α-synuclein protein deposits known as Lewy bodies [1,2,3,4]. Although PD etiology remains unknown, evidence indicates that oxidative stress (OS) plays an essential role in its onset and development, associated with other pathological pathways, including α-synuclein proteostasis, mitochondrial dysfunction, calcium dyshomeostasis, axonal transport dysfunction, and neuroinflammation. Crosstalk between these factors induces increased OS, creating a feedback loop that accelerates neurodegeneration [1,5]. Importantly, current PD therapies are merely symptomatic, highlighting the need for effective treatments able to modify disease progression.

Currently, PD treatments include monoamine oxidase (MAO) inhibitors (selegiline, rasagiline, and safinamide [6]) directed to increasing dopamine levels at the synaptic clef and to reducing OS generated during dopamine metabolism [7]. MAO enzymes (MAO-A and B isozymes) catalyze the oxidative deamination of biological amines including dopamine. Amine metabolism produces high quantities of hydrogen peroxide that decomposes to generate free radicals, thus contributing to an exacerbated OS status that, finally, induces high cytotoxicity under pathological conditions [6]. Additionally, the use of selective MAO-B inhibitors for PD is based on several observations. Human brain postmortem studies have demonstrated a MAO-B levels increase with aging, the main risk factor of PD [8]. Given that MAO-B is mainly present in glial cells, its levels are also augmented as a consequence of gliosis [9]. Thus, astrocytes surrounding dopaminergic neurons at SNpc contribute to the increased OS status due to dopamine metabolism. As previously stated, hydrogen peroxide is produced in this process and it can be transferred to close neurons, which usually contain lower glutathione (GSH) levels than astrocytes to control OS. it makes them more susceptible to this toxic stimulus [10,11]. Additionally, MAO-B is responsible for γ-aminobutyric acid (GABA) synthesis [12]. Released GABA from astrocytes inhibits adjacent dopaminergic neurons at substantia nigra further decreasing dopamine levels [13,14]. Therefore, although currently used MAO-B inhibitors only offer symptomatic relief being unable to modify the progression of the disease, these recent findings support the therapeutic potential of selective MAO-B inhibition.

Regarding the multifactorial nature of PD and its complex pathological network, we considered the use of the intrinsic antioxidant and anti-inflammatory response as a key target for its treatment. In this line, the nuclear factor-erythroid 2 p45-related factor 2 (NRF2) is considered the master regulator of the phase II antioxidant response. It is a transcription factor encoded by *NFE2L2* gene that controls about 1% of human genes preceded by the antioxidant response element (ARE) [15]. It regulates a plethora of processes including inflammation, redox balance, and protein homeostasis [16,17]. Importantly, NRF2 activity decreases during aging the main PD risk factor. Moreover, it has been widely demonstrated that the expression of several NRF2-dependent genes is increased in PD brains (i.e., heme oxygenase 1 (*HMOX1*), glutamate-cysteine ligase regulatory subunit (*GCLM*), sequestosome-1 (*SQSTM*), and NAD(P)H:quinone oxidoreductase 1 (*NQO1*)) [18,19,20,21,22]. This phase II response activation under pathological conditions is an attempt to respond to exacerbated OS. Additionally, NRF2 exhibits nuclear localization in nigral neurons from postmortem samples of PD patients [23] and a positive correlation between its protein levels in cerebral spinal fluid of PD patients and the disease duration or motor scores [24]. Interestingly, in these post-mortem samples, NRF2-dependent proteins NQO1 and p62 were partially trapped in Lewy bodies, suggesting an impaired capacity of the NRF” signature to resolve increased OS status [22]. In addition, there is an association between a protective *NFE2L2* promoter single nucleotide polymorphisms (SNPs) haplotype with a delayed onset and reduced PD risk [21,22,25,26]. Complementing this clinical evidence, NRF2 therapeutic potential for PD treatment is further supported by numerous in vitro and in vivo studies in which NRF2-ARE pathway activation demonstrates protective role in α-synucleinopathy PD models. In this line, ventral midbrain injection of adeno-associated viral vector (rAAV) to express human α-synuclein in *NFE2L2* (^−/−^)-deficient mice induced exacerbated protein aggregation, neuroinflammation, and neuronal death [27]. Moreover, astrocyte-specific *NFE2L2* overexpression significantly reduced synuclein accumulation and provided neuroprotection in the α-synuclein mutant (A53T) mouse model [28]. Interestingly, NRF2 pharmacological activation by dimethyl fumarate (DMF), an approved drug for the treatment of relapsing-remitting multiple sclerosis, reduced dopaminergic neuron loss in the substantia nigra, and decreased astrocytosis and microgliosis in a α-synuclein mouse model [22]. DMF also showed neuroprotection against 6-hydroxydopamine (6-OHDA) [29] and methyl-4-phenyl-1,2,3,6-tetrahydropyridine (MPTP) in in vivo mouse models [30,31,32], being able to modulate microglial dynamics and neuroinflammation [21,33].

Regarding its regulation, under basal conditions cytosolic NRF2 is bound to its repressor protein Kelch like ECH associated protein 1 (KEAP1) that promoted its proteasomal degradation. However, under OS conditions or in the presence of electrophilic compounds, KEAP1 repressor activity is weakened, leading to NRF2 nuclear accumulation [34]. Electrophilic compounds are able to react with key cysteine residues at KEAP1 inducing conformational changes that prevent NRF2 degradation [35,36]. A prominent example of this process is the DMF mechanism of action whose derived metabolite, monomethyl fumarate (MMF), reacts with KEAP1-Cys151 to induce the NRF2-ARE pathway activation [37].

Considering multitarget design, melatonin is a neurohormone with a pleiotropic profile, with remarkable antioxidant properties, able to scavenge free radicals generating several metabolites that can also trap more free radicals. This capacity is known as the antioxidant cascade of melatonin [38]. Moreover, it enhances the expression of antioxidant and downregulates pro-oxidant enzymes [39], leading to an interesting neuroprotective profile [40]. Melatonin has shown protective effects in several PD animal models based on brain injections of 6-OHDA [41,42,43], MPTP [44,45,46,47], rotenone [48,49], or lentiviral vectors encoding mutant human α-synuclein [50]. Clinical trials conducted for PD treatment with melatonin mainly showed effectiveness in controlling sleep disturbances associated with the disease and significant reductions in the unified Parkinson’s disease rating scale (UPDRS) score in certain cases [51,52,53,54,55,56,57]. However, additional clinical studies are needed with doses that allometrically projected to humans are at least 10-times higher than those used in clinics [58].

In view of previously introduced evidences, we hypothesized that the combination of NRF2 inducing activity with selective MAO-B inhibition could result in a beneficial therapeutic approach for PD treatment. To this end, we developed a new family of multitarget compounds considering melatonin structure as a scaffold, given its interesting neuroprotective profile. Structural modifications led to the inclusion of NRF2-ARE pathway induction capacity and, finally, novel compounds were also designed as selective MAO-B inhibitors based on structural similarity with known inhibitors. Here, we described one of the first series of dual NRF2 activators and selective MAO-B inhibitors, as an innovative approach for the development of new PD therapies.

## 2. Materials and Methods

### 2.1. Chemistry

#### 2.1.1. Chemical Methods

All reagents were commercial compounds with high purity. Argon atmosphere was employed for all reactions. Triethylamine (Et_3_N) and dichloromethane (DCM) were dried by distillation over NaOH and CaH_2_, respectively. Acetonitrile (MeCN) was purchased anhydrous. Analytical thin layer chromatography (TLC) was carried out on aluminum plates with Merck Silicagel 60 F254 and visualized by UV irradiation (254 nm). Flash column chromatography was performed with Merck Kieselgel 60 (230–400 mesh). Proton nuclear magnetic resonance (^1^H NMR) spectra were recorded in DMSO at room temperature in a Bruker DRX-300 MHz device. The proton spectra was reported as follows: chemical shifts (δ) in ppm (number of protons, multiplicity, coupling constant J in Hz, assignment). Carbon nuclear magnetic resonance (^13^C NMR) were recorded in DMSO-d6 at room temperature using the same spectrometers at 75 MHz. The infrared spectra (IR) were obtained using an Agilent Technologies Cary 630 FTIR spectrophotometer from a thin film. Purity of the compounds was studied by high-performance liquid chromatography (HPLC), coupled with high-resolution mass spectrometry (HRMS) electrospray with positive mode detection for mass determination, using a mass spectrometer with a quadrupole time-of-fight analyzer (QTOF) model QSTAR pulsar I (Applied Biosystems, Waltham, MA, USA).

#### 2.1.2. Preparation of Tert-butyl-(2-(5-hydroxy-1*H*-indol-3-yl)ethyl)carbamate (**3**)

To a solution of serotonin hydrochloride (1.00 eq) in tetrahydrofuran (THF) under argon, di-tert-butyl dicarbonate (1.10 eq) and Et_3_N (1.10 eq) were added slowly. The solution was stirred under argon for 90 min, quenched with NH_4_Cl, and extracted with ethyl acetate (AcOEt). The organic layers were dried over MgSO_4_ and concentrated under vacuum. The resulting product was purified by flash chromatography on silica gel (Hexane:AcOEt 0–45%) to afford compound **3** as a white solid (99% yield). Experimental results were in agreement with previously reported data [1].

#### 2.1.3. General Procedure for the Synthesis of 3-(2-((Tert-butoxycarbonyl)amino)ethyl)-1*H*-indol-5-yl Cinnamate Derivatives (**6**, **7**)

To a solution of tert-butyl-(2-(5-hydroxy-1*H*-indol-3-yl)ethyl)carbamate (**3**, 1.00 eq) and the corresponding acrylic acid derivative (4 or 5, 1.20 eq) in DCM under argon, N,N’-dicyclohexylcarbodiimide (DCC, 1.20–1.50 eq), HOBt, (0.20 eq), and Et_3_N (1.50 eq) were added. The resulting solution was stirred at room temperature. When completed, the reaction was quenched with distilled water and stirred for 15 min. Then, the mixture was extracted three times with DCM, and washed with 10% aqueous NaHCO_3_ and a saturated solution of NH_4_Cl. The organic layers were dried over MgSO_4_ and filtered. The resulting product was purified by flash chromatography on silica gel using DCM:MeOH mixtures (0–3%) or hexane:AcOEt mixtures (0–80%) as eluent to afford the corresponding 3-(2-((tert-butoxycarbonyl)amino)ethyl)-1*H*-indol-5-yl cinnamate derivative.

#### 2.1.4. General Procedure for the Synthesis of 3-(2-Aminoethyl)-1*H*-indol-5-yl Cinnamate Derivatives (**8**, **9**)

To a solution of the corresponding 3-(2-((tert-butoxycarbonyl)amino)ethyl)-1*H*-indol-5-yl cinnamate derivative (**6** or **7**, 1.00 eq) in DCM under argon, trifluoroacetic acid (TFA, 1.05 eq) was added. The resulting solution was stirred at room temperature. When completed, the reaction was quenched with NaHCO_3_, then extracted with AcOEt and washed with a saturated solution of NH_4_Cl. The organic layers were dried over MgSO_4_ and filtered to yield the corresponding 3-(2-aminoethyl)-1*H*-indol-5-yl cinnamate derivative.

#### 2.1.5. General Procedure for the Synthesis of Amine Derivatives **10**–**15**

To a solution of the appropriate 5-methoxytryptamine derivative (1.00 eq) in MeCN, propargyl bromide (1.00 eq) was added. Et_3_N (1.00 eq) was added in the case of a low reaction rate. The resulting solution was stirred at room temperature upon completion. Once completed, products were purified by flash chromatography on silica gel using DCM/MeOH mixtures 0–5% or petroleum ether/EtOAc; MeOH 0–100%; 0–2.5% as eluent, to yield the desired product.

#### 2.1.6. General Procedure for the Synthesis of Amide Derivatives **16**–**18**

To a solution of DCC (1.15 eq) in dry DCM under argon, 2-propynoic acid (1.15 eq) and the appropriate 5-methoxytryptamine derivative (1.00 eq) were added. The resulting solution was stirred at room temperature. Once completed, the solution was filtered and the solvent was removed by evaporation at low pressure. The resulting products were purified by flash chromatography on silica gel using petroleum ether/EtOAc 0–100% or petroleum ether/DCM; MeOH 0–100%; 0–2.5% to yield the corresponding amide product.

#### 2.1.7. 3-(2-((Tert-butoxycarbonyl)amino)ethyl)-1*H*-indol-5-yl Cinnamate (**6**)

General procedure 2.1.3, tert-butyl-(2-(5-hydroxy-1*H*-indol-3-yl)ethyl)carbamate (**3**, 1.0 g, 3.62 mmol) and cinnamic acid (**4**, 805 mg, 5.43 mmol), DCC (1.12 g, 5.43 mmol), HOBt (98 mg, 0.72 mmol), Et_3_N (700 µL, 5.43 mmol), and DCM (20 mL), 16 h, flash chromatography on silica gel (hexane:EtOAc 0–50%) to afford compound **6** as a white solid (1.17 g, 80% yield). λ_max_ (film)/cm^−1^ 3377, 3301, 3060, 2978, 2936, 1725, 1635, 1580, 1388, 1171, 1142, 1102, 982, 873, 763. ^1^H NMR (300 MHz, DMSO) δ_H_ 10.94 (1H, s_br_, NH), 7.87 (1H, d, J = 16.1 Hz, 3′-H), 7.84–7.80 (2H, m, 2″-H), 7.48–7.44 (3H, m, 32″-H, 42″-H), 7.37 (1H, d, J = 8.6 Hz, 7-H), 7.29 (1H, d, J = 2.2 Hz, 4-H), 7.22 (1H, d, J = 2.3 Hz, 2-H), 6.93–6.88 (2H, m, 2′-H, 6-H), 3.24–3.15 (2H, m, CH_2_CH_2_NHCOO(CH_3_)_3_), 2.79 (2H, t, J = 7.4 Hz, CH_2_CH_2_NHCOO(CH_3_)_3_), 1.38 (9H, S, CH_2_CH_2_NHCOO(CH_3_)_3_). ^13^C NMR (75 MHz, DMSO) δ_C_ 165.7, 155.5, 145.7, 145.5, 143.2, 133.9, 130.6, 128.9, 128.5, 127.4, 124.2, 117.7, 115.3, 112.2, 111.6, 110.3, 77.4, 40.8, 28.2, 25.3. HRMS (ES^+^) mass calculated for C_24_H_26_N_2_O_6_ 406.1893; found [(M + H)^+^] 407.1965; found [(M + Na)^+^] 429.1917.

#### 2.1.8. (E)-3-(2-((Tert-butoxycarbonyl)amino)ethyl)-1*H*-indol-5-yl 3-*p*-tolylacrylate (**7**)

General procedure 2.1.3, tert-butyl-(2-(5-hydroxy-1*H*-indol-3-yl)ethyl)carbamate (**3**, 1.0 g, 3.62 mmol) and (E)-3-(*p*-tolyl)acrylic acid (**5**, 880 mg, 5.43 mmol), DCC (1.12 g, 5.43 mmol), HOBt (98 mg, 0.72 mmol), Et_3_N (700 µL, 5.43 mmol) and DCM (20 mL), 16 h, flash chromatography on silica gel (Hexane:EtOAc 0–50%) to afford **7** as a white solid (1.18 g, 78% yield). λ_max_ (film)/cm^−1^ 3374, 3306, 2977, 2930, 2861, 1720, 1690, 1516, 1455, 1368, 1253, 1169, 991, 815. ^1^H NMR (300 MHz, DMSO) δ_H_ 10.93 (1H, s_br_, NH), 7.83 (1H, d, J = 15.9 Hz, 3′-H), 7.70 (2H, d, J = 7.9 Hz, 32″-H), 7.37 (1H, d, J = 8.6 Hz, 7-H), 7.29-7.27 (3H, m, 4-H, 22″-H), 7.22 (1H, d, J = 2.3 Hz, 2-H), 6.91–6.80 (3H, m, 6-H, 2′-H, NH), 3.22–3.16 (2H, m, CH_2_CH_2_NHCOO(CH_3_)_3_), 2.79 (2H, t, J = 7.4 Hz, CH_2_CH_2_NHCOO(CH_3_)_3_), 2.36 (3H, s, CH_3_-Ph) 1.38 (9H, s, CH_2_CH_2_NHCOO(CH_3_)_3_). ^13^C NMR (75 MHz, DMSO-d_6_) δ_C_ 165.8, 155.5, 145.7, 143.2, 140.7, 133.9, 131.2, 129.5, 128.5, 127.4, 124.1, 116.5, 115.3, 112.2, 111.6, 110.3, 77.3, 40.8, 28.2, 25.3, 21.0. HRMS (ES^+^) mass calculated for C_25_H_28_N_2_O_4_ 420.2049; found [(M + H)^+^] 421.2126; found [(M + Na)^+^] 443.1931.

#### 2.1.9. 3-(2-Aminoethyl)-1*H*-indol-5-yl Cinnamate (**8**)

General procedure 2.1.4, 3-(2-((tert-butoxycarbonyl)amino)ethyl)-1*H*-indol-5-yl cinnamate (**6**, 300 mg, 0.738 mmol), TFA (59.0 µL, 0.775 mmol) in dry DCM (10 mL), 24 h to afford compound **8** as a yellow-orange solid (60% yield)). ^1^H NMR (300 MHz, DMSO) δ_H_ 11.03 (1H, s_br_, NH), 7.90–7.78 (3H, m, 3′-H, 22″-H), 7.47 (3H, m, 32″-H, 42″-H), 7.41–7.33 (2H, m, 7-H, 4-H), 7.27 (1H, s, 2-H), 6.94–6.87 (2H, m, 2′-H, 6-H), 3.02–2.91 (2H, m, CH_2_CH_2_NH_2_), 2.91–2.82 (2H, m, CH_2_CH_2_NH_2_), 1.87 (2H, d, J = 4.9 Hz, NH_2_). ^13^C NMR (75 MHz, DMSO) δ_C_ 168.4, 148.5, 146.0, 136.8, 136.6, 133.4, 131.66, 131.2, 129.8, 127.4, 120.33, 118.1, 114.5, 113.6, 113.1, 28.1, 24.3. HPLC-HRMS (ES^+^) calculated mass for C_19_H_18_N_2_O_2_ 306.1368; found [(M + H)^+^] 307.1440; found [(M + 2H)^+^] 308.1473; found [(2M + H)^+^] 613.2801.

#### 2.1.10. 3-(2-Aminoethyl)-1*H*-indol-5-yl (E)-3-(*p*-tolyl)acrylate (**9**)

General procedure 2.1.4, (3-(2-((tert-butoxycarbonyl)amino)ethyl)-1*H*-indol-5-yl (E)-3-(*p*-tolyl)acrylate (**7**, 300 mg, 0.714 mmol), TFA (57.0 µL, 0.750 mmol) in dry DCM (10 mL), 24 h to afford compound **9** as a yellow solid (90% yield). ^1^H NMR (300 MHz, DMSO) δ_H_ 11.08 (1H, s_br_, NH), 7.82 (1H, d, J = 15.9 Hz, 3′-H), 7.70 (2H, d, J = 8.2 Hz, 22″-H), 7.43–7.33 (2H, m, 7-H, 4-H), 7.32–7.24 (3H, m, 3”H, 2-H), 6.91 (1H, dd, J = 8.6, J = 2.4 Hz, 6-H), 6.83 (1H, d, J = 16.0 Hz, 2′-H), 3.32 (2H, s_br_, NH_2_), 3.05 (2H, t, J = 7.6 Hz, CH_2_CH_2_NH_2_), 2.94 (2H, t, J = 7.7 Hz, CH_2_CH_2_NH_2_), 2.36 (3H, s, Ph-CH_3_); ^13^C NMR (75 MHz, DMSO) δ_C_ 168.5, 148.6, 146.1, 143.5, 136.8, 134.0, 132.3, 131.3, 129.6, 127.6, 119.1, 118.3, 114.6, 113.0, 112.5, 42.4, 26.2, 23.8. HPLC-HRMS (ES^+^) calculated mass for C_20_H_20_N_2_O_2_ 320.1525; found [(M + H)^+^] 321.1606; found [(M + 2H)^+^] 322.1613.

#### 2.1.11. *N*-(2-(5-Methoxy-1*H*-indol-3-yl)ethyl)prop-2-yn-1-amine (**10**)

General procedure 2.1.5, 5-methoxytryptamine (50 mg, 0.263 mmol) in MeCN (8 mL), propargyl bromide (29.3 µL, 0.263 mmol), 18 h, flash chromatography on silica gel (DCM:MeOH 0–3%) to afford compound **10** as a red oil (70% yield). λ_max_ (film)/cm^−1^ 3413, 3279, 2919, 2829, 1587, 1483, 1216, 797, 643. ^1^H NMR (300 MHz, DMSO) δ_H_ 10.61 (1H, s_br_, NH), 7.22 (1H, d, J = 8.7 Hz, d, 7-H), 7.09 (1H, d, J = 2.3 Hz, 2-H), 7.00 (1H, d, J = 2.3 Hz, 4-H), 6.71 (1H, dd, J = 8.7, J = 2.4 Hz, 6-H), 3.76 (3H, s, O-CH_3_), 3.41–3.27 (3H, m, CH_2_CCH, CH_2_CH_2_NH), 3.04 (1H, t, J = 2.3, CH_2_CCH), 2.88–2.73 (4H, m, CH_2_CH_2_NH, CH_2_CH_2_NH); ^13^C NMR (75 MHz, DMSO) δ_C_ 152.8, 131.3, 127.5, 123.2, 112.2, 111.9, 110.9, 100.1, 83.1, 73.4, 55.3, 48.6, 37.3, 25.1. HPLC-HRMS (ES^+^) calculated mass for C_14_H_16_N_2_O 228.1273; found [(M + H)^+^] 229.1325; purity 96% (HPLC).

#### 2.1.12. 3-(2-(Prop-2-yn-1-ylamino)ethyl)-1*H*-indol-5-yl Cinnamate (**11**)

General procedure 2.1.5, 3-(2-aminoethyl)-1*H*-indol-5-yl cinnamate (107 mg, 0.349 mmol) in MeCN (10 mL), propargyl bromide (51.9 µL, 0.349 mmol) and Et_3_N (46.3 µL, 0.349 mmol) were added, 72 h, flash chromatography on silica gel (petroleum ether:EtOAc 0–100%:MeOH 0–3%) to afford compound **11** as a yellow solid (31% yield). λ_max_ (film)/cm^−1^ 3434, 3257, 2918, 1626, 1472, 1388, 1299, 1212, 1038, 971. ^1^H NMR (300 MHz, DMSO) δ_H_ 8.40 (1H, d, J = 9.0 Hz, 7-H), 8.24 (1H, s, 2-H), 7.99–7.88 (6H, m, 22″-H, 3′-H, NH, NH_2_^+^), 7.73 (1H, d, J = 15.4 Hz, 2′-H), 7.53–7.47 (3H, m, 32″-H,42″-H), 7.27 (1H, d, J = 2.5 Hz, 4-H), 7.05 (1H, dd, J = 8.9, J = 2.6 Hz, 6-H), 4.88 (2H, d, J = 2.4 Hz, CH_2_CCH), 3.57 (1H, t, J = 2.4 Hz, CH_2_CCH), 3.24–3.16 (2H, m, 2H, CH_2_CH_2_NH), 3.00 (2H, t, J = 7.5 Hz, CH_2_CH_2_NH). ^13^C NMR (75 MHz, DMSO) δ_C_ 163.9, 154.5, 146.2, 134.8, 131.6, 131.2, 131.1, 129.4, 129.3, 125.4, 118.2, 117.7, 117.4, 114.1, 104.2, 79.8, 78.6, 56.4, 38.6, 23.2. HPLC-HRMS (ES^+^) calculated mass for C_22_H_20_N_2_O_2_ 344.1525; found [(M + H)^+^] 345.1595; [(2 M + H)^+^] 689.3136; purity 96% (HPLC).

#### 2.1.13. 3-(2-(Prop-2-yn-1-ylamino)ethyl)-1*H*-indol-5-yl-(E)-3-(*p*-tolyl)acrylate (**12**)

General procedure 2.1.5, 3-(2-aminoethyl)-1*H*-indol-5-yl-(E)-3-(*p*-tolyl) acrylate (95.0 mg, 0.296 mmol) in MeCN (10 mL), propargyl bromide (92.9 µL, 0.296 mmol) and Et_3_N (40 µL, 0.296 mmol) were added, 72 h, flash chromatography on silica gel (petroleum ether:EtOAc 0–100%:MeOH 0–3%) to afford compound **12** as a yellow solid (30% yield). λ_max_ (film)/cm^−1^ δ 3360, 3258, 3082, 2923, 2861, 1671, 1606, 1473, 1452, 1387, 1252, 1217, 1052, 1024, 973, 815. ^1^H NMR (300 MHz, DMSO) δ_H_ 8.37 (1H, d, J = 9.0 Hz, 7-H), 8.15 (s, 1H, 2-H), 7.84 (3H, m, 3′-H, 22″-H), 7.69 (1H, d, J = 15.3 Hz, 2′-H), 7.30 (2H, d, J = 8.2 Hz, 32″-H), 7.21 (1H, d, J = 2.5 Hz, 4-H), 6.99 (1H, dd, J = 9.0, J = 2.6 Hz, 6-H), 4.86 (2H, d, J = 2.4 Hz, CH_2_CCH), 3.58 (1H, s, J = 2.2 Hz, CH_2_CCH), 2.91 (2H, t, J = 6.6 Hz, CH_2_CH_2_NH), 2.75 (2H, J = 6.6 Hz, CH_2_CH_2_NH), 2.37 (3H, s, O-CH3), 1.94 (1H, sbr, 1H, CH_2_CH_2_NH). ^13^C NMR (75 MHz, DMSO) δ_C_ 163.9, 154.3, 145.9, 141.0, 132.2, 132.1, 130.9, 129.8, 129.3, 124.4, 120.2, 117.6, 117.1, 113.8, 104.0, 79.8, 78.5, 56.2, 41.7, 29.3, 21.4. HPLC-HRMS (ES^+^) calculated mass for C_23_H_22_N_2_O_2_ 358.1681; found [(M + H)^+^] 359.1845; [(2 M + H)^+^] 717.3441; purity 96% (HPLC).

#### 2.1.14. *N*-(2-(5-Methoxy-1*H*-indol-3-yl)ethyl)-*N*-(prop-2-yn-1-yl)prop-2-yn-1-amine (**13**)

General procedure 2.1.5, 5-methoxytryptamine (50 mg, 0.263 mmol) in MeCN (8 mL), propargyl bromide (29.3 µL, 0.263 mmol), 18 h, flash chromatography on silica gel (petroleum ether: EtOAc 0–100%: MeOH 0–2.5%) to afford compound **4** as a red oil (57% yield). λ_max_ (film)/cm^−1^ 3248, 3173, 2932, 2827, 1486, 1435, 1215, 1069, 790, 676, 657. ^1^H NMR (300 MHz, DMSO) δ_H_ 10.59 (1H, s_br_, NH), 7.21 (1H, d, J = 8.7 Hz, d, 7-H), 7.09 (1H, d, J = 2.4 Hz, 2-H), 7.00 (1H, d, J = 2.4 Hz, 4-H), 6.69 (1H, dd, J = 8.8, J = 2.5 Hz, 6-H), 3.75 (3H, s, O-CH_3_), 3.45 (4H, d, J = 2.3 Hz, CH_2_CCH), 3.16 (2H, d, J = 2.3, CH_2_CCH), 2.82–2.68 (4H, m, CH_2_CH_2_NH, CH_2_CH_2_NH); ^13^C NMR (75 MHz, DMSO) δ_C_ 152.9, 131.3, 127.4, 123.2, 111.9, 111.8, 111.0, 100.0, 79.3, 75.5, 55.3, 52.8, 41.6, 23.0. HPLC-HRMS (ES^+^) calculated mass for C_17_H_18_N_2_O 266.1419; found [(M + H)^+^] 267.1490; purity 95% (HPLC).

#### 2.1.15. 3-(2-(Di(prop-2-yn-1-yl)amino)ethyl)-1*H*-indol-5-yl Cinnamate (**14**)

General procedure 2.1.5, 3-(2-aminoethyl)-1*H*-indol-5-yl cinnamate (107 mg, 0.349 mmol) in MeCN (10 mL), propargyl bromide (51.9 µL, 0.349 mmol) and Et_3_N (46.3 µL, 0.349 mmol) were added, 72 h, flash chromatography on silica gel (petroleum ether:EtOAc 0–100%:MeOH 0–3%) to afford compound **14** as a yellow oil (39% yield). λ_max_ (film)/cm^−1^ 3276, 2926, 2846, 1709, 1628, 1449, 1240, 1203, 1171, 955, 771. ^1^H NMR (300 MHz, DMSO) δ_H_ 10.90 (1H, s_br_, NH), 7.89–7.74 (3H, m, 2′-H, 22″-H), 7.50–7.42 (3H, m, 32″-H, 42″-H), 7.35 (1H, d, H-7), 7.31 (1H, d, J = 2.3 Hz, 2-H), 7.23 (1H, d, J = 2.3 Hz, 4-H), 6.95–6.82 (2H, m, 3′-H, 6-H), 3.45 (4H, d, J = 2.4 Hz, CH_2_CCH), 3.13 (2H, t, J = 2.2 Hz, CH_2_CCH), 2.86–2.68 (4H, m, CH_2_CH_2_N, CH_2_CH_2_N); ^13^C NMR (75 MHz, DMSO) δ_C_ 168.4, 148.4, 145.9, 136.7, 133.4, 131.7, 131.2, 130.0, 126.8, 120.4, 117.9, 115.1, 114.3, 113.1, 82.0, 78.2, 55.6, 44.2, 36.0, 25.5. HPLC-HRMS (ES+) calculated mass for C_25_H_22_N_2_O_2_ 382.1681; found [(M + H)^+^] 383.1752; purity 95% (HPLC).

#### 2.1.16. 3-(2-(Di(prop-2-yn-1-yl)amino)ethyl)-1*H*-indol-5-yl-(E)-3-(*p*-tolyl) Acrylate (**15**)

General procedure 2.1.5, 3-(2-aminoethyl)-1*H*-indol-5-yl-(E)-3-(*p*-tolyl) acrylate (95.0 mg, 0.296 mmol) in MeCN (10 mL), propargyl bromide (92.9 µL, 0.296 mmol) and Et_3_N (40 µL, 0.296 mmol) were added, 72 h, flash chromatography on silica gel (petroleum ether:EtOAc 0–100%:MeOH 0–2.5%) to afford compound **15** as a pale-yellow oil (41% yield). λ_max_ (film)/cm^−1^ 3309, 3255, 3181, 2926, 2852, 2834, 1725, 1641, 1460, 1312, 1168, 1147, 979, 810. ^1^H NMR (300 MHz, DMSO) δ_H_ 10.95 (1H, s_br_, NH), 7.86 (1H, d, J = 16.0 Hz, 2′-H), 7.74 (2H, d, J = 8.1 Hz, 22″-H), 7.39 (1H, d, J = 8.7 Hz, 7-H), 7.36–7.29 (3H, m, 32″-H, 2-H), 7.27 (1H, d, J = 2.3 Hz, 4-H), 6.97–6.80 (2H, m, 3′-H, 6-H), 3.49 (4H, d, J = 2.4 Hz, CH_2_CCH), 3.17 (2H, t, J = 2.3 Hz, CH2CCH), 2.92–2.72 (4H, m, CH_2_CH_2_N, CH_2_CH_2_N), 2.40 (3H, s, Ph-CH_3_); ^13^C NMR (75 MHz, DMSO) δ_C_ 165.8, 145.7, 143.2, 140.7, 133.9, 131.3, 129.6, 128.5, 127.3, 116.6, 115.3, 112.4, 111.6, 110.4, 79.2, 75.5, 53.0, 41.5, 22.8, 21.0. HPLC-HRMS (ES^+^) calculated mass for C_26_H_24_N_2_O_2_ 396.1838; found [(M + H)^+^] 397.1909; [(2M + H)^+^] 793.3738; purity 95% (HPLC).

#### 2.1.17. *N*-(2-(5-Methoxy-1*H*-indol-3-yl)ethyl) Propiolamide (**16**)

General procedure 2.1.6, DCC (281 mg, 1.36 mmol), 2-propynoic acid (88.1 µL, 1.36 mmol) in dry DCM (10 mL), 5-methoxytryptamine (225 mg, 1.18 mmol), 24 h, flash chromatography on silica gel (petroleum ether: DCM 0–100%:methanol (MeOH) 0–3%) to afford compound **7** as a pale red oil (45% yield). λ_max_ (film)/cm^−1^ 3373, 3262, 2931, 2111, 1642, 1535, 1488, 1275, 1216, 1032, 802. ^1^H NMR (300 MHz, DMSO) δ_H_ 10.68 (1H, sbr, NH), 8.84 (1H, s_br_, NHC(O)CCH), 7.26 (1H, d, J = 8.7 Hz, d, 7-H), 7.14 (1H, d, J = 2.4 Hz, 2-H), 7.05 (1H, d, J = 2.5 Hz, 4-H), 6.75 (1H, dd, J = 8.7, J = 2.4 Hz, 6-H), 4.12 (1H, s, NHC(O)CCH), 3.80 (3H, s, O-CH_3_), 3.47–3.32 (2H, m, CH_2_CH_2_NH), 2.85 (2H, t, J = 7.4, CH_2_CH_2_NH); ^13^C NMR (75 MHz, DMSO) δ_C_ 153.0, 151.6, 131.3, 127.5, 123.3, 111.9, 111.2, 111.0, 100.1, 78.5, 75.3, 55.3, 39.7, 24.6. HPLC-HRMS (ES+) calculated mass for C_14_H_14_N_2_O_2_ 242.1055; found [(M + H)^+^] 243.1153; [(2M + H)^+^] 485.2179; [(2M + Na)^+^] 507.2001; purity 100% (HPLC).

#### 2.1.18. 3-(2-Propiolamidoethyl)-1*H*-indol-5-yl Cinnamate (**17**)

General procedure 2.1.6, DCC (58.1 mg, 0.281 mmol), 2-propynoic acid (17.2 µL, 0.281 mmol) in dry DCM (8 mL), 3-(2-aminoethyl)-1*H*-indol-5-yl cinnamate (75.0 mg, 0.244 mmol), 5 h, flash chromatography (petroleum ether:EtOAc 0–100%) to afford compound **17** as a white solid (89% yield). λ_max_ (film) / cm^−1^ 3386, 3299, 3224, 3054, 2936, 2107, 1713, 1642, 1547, 1168, 1031, 766. ^1^H NMR (300 MHz, DMSO) δ_H_ 10.94 (1H, s_br_, NH), 8.80 (1H, s_br_, NHC(O)CCH), 7.92–7.75 (3H, m, 2′-H, 22″-H), 7.53–7.41 (3H, m, 32″-H, 42″-H), 7.36 (1H, d, J = 8.7 Hz, 7-H), 7.30 (1H, d, J = 2.2 Hz, 2-H), 7.22 (1H, d, J = 2.4 Hz, 4-H), 6.96–6.82 (2H, m, 3′-H, 6-H), 4.07 (1H, s, NHC(O)CCH), 3.43–3.27 (2H, m, CH_2_CH_2_NH), 2.81 (2H, t, J = 7.4 Hz, CH_2_CH_2_NH); ^13^C NMR (75 MHz, DMSO) δ_C_ 165.7, 151.6, 145.8, 143.3, 134.0, 130.7, 129.0, 128.5, 127.2, 124.3, 117.7, 115.4, 111.7, 110.3, 75.4, 40.1, 24.5. HPLC-HRMS (ES^+^) calculated mass for C_22_H_18_N_2_O_3_ 358.1317; found [(M + H)^+^] 359.1389; purity 100% (HPLC).

#### 2.1.19. 3-(2-Propiolamidoethyl)-1*H*-indol-5-yl-(E)-3-(*p*-tolyl) Acrylate (**18**)

General procedure 2.1.6, DCC (55.5 mg, 0.269 mmol), 2-propynoic acid (17.4 µL, 0.269 mmol) in dry DCM (8 mL), 3-(2-aminoethyl)-1*H*-indol-5-yl-(E)-3-(*p*-tolyl) acrylate (75.0 mg, 0.234 mmol), 5 h, flash chromatography (petroleum ether: EtOAc 0–100%) to afford compound **18** as a white solid (78% yield). λ_max_ (film)/cm^−1^ 3270, 2925, 2106, 1717, 1642, 1171, 1147, 816. ^1^H NMR (300 MHz, DMSO) δ_H_ 10.93 (1H, s_br_, NH), 8.80 (1H, s_br_, NHC(O)CCH), 7.81 (1H, d, J = 16.0 Hz, 2′-H), 7.69 (2H, d, J = 7.8 Hz, 22″-H), 7.35 (1H, d, J = 8.5 Hz, d, 7-H), 7.29 (2H, d, J = 2.3 Hz, 3-H”), 7.26 (1H, s, 2-H), 7.22 (1H, m, H-4), 6.93–6.75 (2H, m, 3′-H, 6-H), 4.07 (1H, s, NHC(O)CCH), 3.44–3.19 (2H, m, CH_2_CH_2_NH), 2.81 (2H, t, J = 7.4 Hz, CH_2_CH_2_NH), 2.35 (3H, s, Ph-CH_3_); ^13^C NMR (75 MHz, DMSO) δ_C_ 165.8, 151.5, 145.7, 143.3, 140.7, 134.0, 131.3, 129.6, 128.5, 127.2, 116.5, 115.4, 111.7, 110.3, 78.4, 75.4, 39.6, 24.5, 21.0. HPLC-HRMS (ES+) calculated mass for C_23_H_20_N_2_O_3_ 373.1507; found [(M + H)^+^] 372.1474; [(2M + H)^+^] 745.3009; purity 100% (HPLC).

### 2.2. Biological Evaluation

#### 2.2.1. AREc32 Cell Line Culture

AREc32 cells, shared by CR Wolf, were grown in Dulbecco’s modified Eagle medium (DMEM) with GlutaMAX (Gibco, Invitrogen, Madrid, Spain), supplemented with 10% (*v*/*v*) filtrated fetal bovine serum (FBS; Gibco, Invitrogen, Madrid, Spain), 1% of antibiotics penicillin-streptomycin, and geneticin (0.8 mg/mL G418; Gibco, Invitrogen, Madrid, Spain). Cells were maintained at 37 °C under humidified atmosphere (5% CO_2_ and 95% relative humidity). Cells were cultured in flasks (Corning, Madrid, Spain) until reaching 80% confluence and sub-cultured using 0.25% EDTA-trypsin (Thermo Fisher, Madrid, Spain) for 5 min. They were recovered by centrifugation at 800 rpm for 10 min. Cells were used from the third to twelfth passage.

#### 2.2.2. Luciferase Activity: NRF2 Induction

AREc32 cells were stably transfected with the plasmid pGL-8xARE that implements 8 copies of the antioxidant response element (ARE) sequences followed by luciferase reporter gen. Therefore, NRF2 induction can be related to the activation of ARE sequences by measuring luciferase activity in terms of luminescence production. For NRF2 induction experiments, cells were plated in 96 white flat bottom well plates (20,000 cells/well). After 24 h, cells were incubated with selected compounds at the desired concentrations in duplicates for 24 h. The Luciferase Assay System (Promega E1500) was used according to provider protocol and luminescence was quantified in an Orion II microplate luminometer (Berthold, Germany). Fold induction of luciferase activity was normalized to basal conditions. Data were expressed as CD values, which means the concentration needed to duplicate the luciferase activity compared to basal conditions. CD values were calculated from dose–response curves fitted by non-linear regression for each compound after logarithmic transformation of the data using GraphPad Prism 8.0 software.

#### 2.2.3. Inhibition of Monoamine Oxidase Enzymes (MAO-A and MAO-B)

Assays were performed in 96 black flat bottom well plates in a final volume of 200 µL/well. Test compounds at the desired concentrations were pre-incubated with human recombinant monoamine oxidase enzymes hrMAO-A or hrMAO-B (Sigma–Aldrich, Madrid, Spain) at final concentrations of 0.0180 or 0.0135 U/mL, respectively, during 30 min at 37 °C. Reaction was started by adding Amplex UltraRed reagent (12.5 µM final concentration; Invitrogen, Madrid, Spain), horseradish peroxidase (0.02 U/mL final concentration; Sigma–Aldrich, Spain), and tyramine (0.5 mM final concentration; Sigma–Aldrich, Spain). Resorufin production was measured at 530/590 nm (excitation/emission) during 30 min in a FluoStar Optima (BMG Labtech, Ortenberg, Germany) fluorescence plate reader. All solutions were prepared in 25 mM of sodium phosphate buffer pH 7.4. Clorgyline (MAO-A selective inhibitor) and rasagiline (MAO-B selective inhibitor) were used as positive controls of inhibition. IC_50_ values for MAO-A and MAO-B inhibition were calculated from dose–response curves obtained by non-linear regression fitting after logarithmic transformation of the data using GraphPad Prism 8.0 software.

#### 2.2.4. MAO-A and MAO-B Reversibility Assays

Assays were performed following the general procedure 2.15: Inhibition of monoamine oxidase enzymes (MAO-A and MAO-B), modifying in this case the pre-incubation time. Briefly, 0-, 15-, and 30-min pre-incubation times were used to obtain MAO inhibition activities versus time curves. MAO inhibition activity varies over time for irreversible inhibitors; however, it remains stable for reversible type inhibitors. Rasagiline and clorgyline were used as references of irreversible inhibition for comparative purposes and each compound was incubated at a 1.8xIC_50_-μM final concentration to observe adequate MAO inhibition.

#### 2.2.5. Oxygen Radical Absorbance Capacity (ORAC) Assay

The ORAC test [59] was performed to evaluate the oxygen free radical scavenger capacity of the compounds. (±)-6-hydroxy-2,5,7,8-tetramethylchromane-2-carboxylic acid (Trolox) at 1, 2, 4, 6, and 8 µM concentrations was used as reference compound and melatonin was used as positive control. Compounds at desired concentrations (0.03, 0.1, 0.3, 1, 3, and 10 µM) were diluted in phosphate-buffered saline (PBS; 10 mM, pH 7.4) at 37 °C and placed in a 96 black flat bottom well plate. Then 150 µL of fluorescein (70 nM final concentration), 25 µL of PBS buffer for blank, 25 µL of Trolox solution for standard, and 25 µL of melatonin or compound were added to each well. A fluorescence measurement was done first to determine the basal signal. Then, 25 µL of 2, 2′-azobis-amidinopropane dihydrochloride (AAPH) (12 mM final concentration) was quickly added. All samples were measured in duplicates in three different experiments. Fluorescence was recorded at 485/520 nm (excitation/emission) for 90 min at 37 °C to obtain the area under the fluorescence decay curve (AUC) in a FLUOstar Optima plate reader (BMG Labtech, Ortenberg, Germany). Results were expressed in terms of Trolox equivalents (T.eq.), obtained by plotting net AUC versus compound concentration, then performing linear regression and normalizing slopes of the compounds considering Trolox as reference.

#### 2.2.6. Blood–Brain Barrier Permeation Assay (PAMPA)

Prediction of the compounds’ ability to cross the blood–brain barrier (BBB) by passive diffusion was evaluated using the parallel artificial membrane permeation assay (PAMPA), as described previously [60]. Briefly, permeability of the compounds was measured at an initial concentration of 100 µM, including positive and negative controls (Supplementary Data; Table S3). To model the BBB, the filter membrane of the 96-well donor plate (multiscreen IP sterile clear plate PDVF membrane, pore size 0.45 μM) was filled with 4 µL of porcine brain lipid (PBL, Avanti Polar Lipids, Inc., Birmingham, UK) dissolved in dodecane (20 mg/mL, Sigma–Aldrich, Madrid, Spain). After 5 min, 180 µL of each compound solution in PBS (10 mM, pH 7.4) was added to determine their ability to pass the membrane (V_D_). The 96-well acceptor plate (Multiscreen, Millipore Corp., Madrid Spain) was loaded with 180 µL of PBS (V_A_). Then, the donor filter plate was carefully placed on the acceptor plate to form a sandwich-like system, which was left undisturbed for 4 h at room temperature. After incubation, the donor plate was removed and the absorbance at the maximum absorption wavelength for each compound was recorded in both the acceptor and donor wells (150 µL/well) using a UV plate reader SPECTROstar Nano (BMG Labtech, Ortenberg, Germany). Concentration of the compounds in the donor and acceptor plates and equilibrium concentration were calculated and expressed as permeability (Pe) according the Equation (1). Results are expressed as mean ± standard error of the mean (SEM) of at least three different experiments in duplicate:

(1) $$\log\mathrm{Pe}\;=\log\left\{C\times -\ln\left(1-\frac{{\left[\mathrm{drug}\right]}_{\mathrm{acceptor}}}{{\left[\mathrm{drug}\right]}_{\mathrm{equilibrium}}}\right)\right\}$$

where $C=\left(\frac{V_{D}\times V_{A}}{\left(V_{D\;}+V_{A}\right)\times \mathrm{Area}\times \mathrm{Time}}\right)$.

#### 2.2.7. SH-SY5Y Neuroblastoma Cell Line Culture

SH-SY5Y human neuroblastoma cell line (ECACC, 94030304) was grown in a modified minimum essential medium (MEM) (4.765 g/L MEM; 2.5% minimum essential medium–non-essential amino acids (Invitrogen, Madrid, Spain); Ham’s F12 Nutrients Mix (Thermo Fisher, Massachusetts, EEUU); 0.5 mM of sodium pyruvate (Sigma–Aldrich, Madrid, Spain); 2 g/L of NaHCO_3_ (PanReac, Barcelona, Spain); 10% (*v*/*v*) filtrated FBS (Gibco, Invitrogen, Madrid, Spain), and 100 U/mL of penicillin/streptomycin (Invitrogen, Spain)). Cells were maintained at 37 °C under humidified atmosphere (5% CO_2_ and 95% relative humidity). Cells were cultured in flasks (Corning, Madrid, Spain) until reaching 80% confluence and sub-cultured using 0.25% EDTA-trypsin (Thermo Fisher, Madrid, Spain) for 5 min. They were recovered by centrifugation at 800 rpm for 10 min. Cells from the third to twelfth passage were seeded at a density of 80,000 cells/well in 96-well plates for the neuroprotection studies.

#### 2.2.8. Neuroprotection Studies in the SH-SY5Y Neuroblastoma Cell Line

Cells were seeded at a density of 60,000 cells/well in 96-well transparent plates for 24 h. Then cells were pre-incubated with the corresponding compound at 0.1 μM. After 24 h, the medium was removed and replaced with 1% (*v*/*v*) FBS media containing the corresponding compound at 0.1 µM and the selected toxic stimuli, namely a mixture of rotenone and oligomycin A (R/O; 30/10 μM, respectively; Sigma–Aldrich, Spain) or 6-hydroxydopamine (100 µM; Sigma–Aldrich, Spain). The co-incubation of toxic stimuli plus compounds was maintained for 24 h. Melatonin and rasagiline were used as references for comparative purposes. After the co-incubation period, cell viability was assessed by the 3-(4,5-dimethylthiazol-2-yl)-2,5-diphenyltetrazolium bromide (MTT) method considering basal conditions as 100% survival.

#### 2.2.9. Ethics for Animal Experimentation

All experimental procedures were performed following the Guide for the Care and Use of Laboratory Animals, in accordance with the European Guidelines for the use and care of animals for research in accordance with the European Union Directive of 22 September 2010 (2010/63/UE) and with the Spanish Royal Decree of 1 February 2013 (53/2013).

#### 2.2.10. Mixed Primary Glial Culture

Mixed primary glial cultures were obtained from cerebral cortex of 2–5-day-old Sprague–Dawley rats. Blood vessels and meninges were first removed in Hank’s balanced salt solution (HBSS) and then forebrains were dissociated in DMEM/F12 medium. After mechanical dissociation, filtering, and centrifugation at 800 rpm for 10 min, cells were plated at a density of 30,000 cells/well in DMEM/F12 medium with 20% (*v*/*v*) FBS and 1% penicillin/streptomycin (10,000 units). Cells were maintained at 37 °C under humidified atmosphere (5% CO_2_ and 95% relative humidity). After 5 days, medium was substituted by DMEM/F12 medium with 10% (*v*/*v*) FBS. Cells were cultured for 7–10 days before treatment.

#### 2.2.11. Nitrite Production Measurement in Mixed Primary Glial Cultures

Primary glial cells were pre-treated with compounds at the desired concentration for 24 h. Then, treatments were removed and glial cells were incubated with lipopolysaccharide (LPS, 1 μg/mL; Sigma–Aldrich, Spain) in the presence of each compound at the desired concentration for 18 h. Nitrite production was then assessed by modified Griess assay [61]. Briefly, 150 µL of each sample was mixed with 75 µL of 4,4′-diamino-diphenylsulfone (Dapsone) and 75 µL of *N*-(1-naphthyl)ethylenediamine (Neda). The mixture was incubated at room temperature for 5 min. Then, absorbance was measured at 550 nm in a microplate reader SPECTROstar Nano (BMG Labtech, Ortenberg, Germany). Data were normalized to basal conditions, setting this value as 100% of nitrite production. EC_50_ values were calculated from dose–response curves obtained from representing percentage of nitrites reduction vs. compound concentration.

#### 2.2.12. Quantification of IL-1β Levels by ELISA

Supernatants obtained from mixed primary glial cell cultures were used to determine IL-1β levels by quantitative ELISA kits (Preprotech, R&D Systems-bioNova, Madrid, Spain) following the manufacturer’s instructions. Commercial solution of IL-1β was used to generate a standard curve for sample quantification. Absorbance was measured in a microplate reader SPECTROstar Nano (BMG Labtech) at 405 with 650 nm correction.

#### 2.2.13. Acute Treatment of Rat Striatal Slices

Three- to four-month-old Sprague–Dawley rats were decapitated, the brain was removed, and the two brain hemispheres were separated. Each hemisphere was then cut into 300-µm coronal slices using a McIlwain Tissue Chopper (Cavey Laboratory Engineering, Surrey, UK) and separated in a previously oxygenated (95% O_2_ and 5% CO_2_) ice-cold Krebs’s dissection buffer (120 mM of NaCl; 2 mM of KCl; 26 mM of NaHCO_3_; 1.18 mM of KH_2_PO_4_; 10 mM of MgSO_4_; 0.5 mM of CaCl_2_; 11 mM of glucose; and 200 mM sucrose at pH 7.4) using a Leica SE6 microscope (Leica, Madrid, Spain). Striatum-containing slices were selected and immediately incubated for stabilization in dissection buffer without sucrose, bubbled with 95% O_2_ and 5% CO_2_ for 45 min at 34 °C, allowing slices to recover from slicing injury. Afterwards, brain slices were placed in a 24-well plate containing new medium composed of 1:1 control buffer (120 mM of NaCl; 2 mM of KCl; 26 mM of NaHCO_3_; 1.18 mM of KH_2_PO_4_; 1.19 mM of MgSO_4_; 2 mM of CaCl_2_; and 11 mM of glucose) and DMEM/F12 medium (Invitrogen, Madrid, Spain). Slices were exposed for 4 h to 6-OHDA (100 μM) or rotenone (10 μM), and co-incubated with corresponding compounds (10 µM). Saline was used instead of toxics for basal conditions. Finally, cell death and the ROS production were measured.

#### 2.2.14. Measurement of Cell Death and ROS Production in Rat Striatal Slices

ROS quantification was performed using the fluorescent probe 2,7′-dichlorodihydrofluorescein diacetate (H_2_DCFDA, λ_Ex/Em_ = 495/529 nm; Thermo Fisher, Madrid, Spain). Cell death quantification was performed using the fluorescent probe propidium iodide (PI, λ_Ex/Em_ = 535/617 nm; Thermo Fisher, Madrid, Spain). Brain slices were incubated with both probes for 45 min at a concentration of 10 µL/mL for H_2_DCFDA and 1 µL/mL for PI, in the presence of 1 µL/mL of Hoechst at 37 °C. Fluorescence from striatum slices was then recorded for H_2_DCFDA (λ_Ex/Em_ = 495/529 nm), PI (λ_Ex/Em_ = 535/617 nm), and Hoechst (λ_Ex/Em_ = 350/461 nm), in an inverted Nikon Eclipse T2000-U microscope (Nikon, Tokyo, Japan). NIS-Elements BR 4.10.04 64-bit software was used to analyze the images.

#### 2.2.15. MEF Cell Lines Culture

Mouse embryonic fibroblasts (MEFs) obtained from NRF2^+/+^ (wild-type; WT) and NRF2^−/−^ (knock-out; KO) mice [62] were grown in DMEM with GlutaMAX (Gibco, Invitrogen, Spain), supplemented with 10% (*v*/*v*) filtrated fetal bovine serum (FBS; Gibco, Invitrogen, Madrid, Spain), and 1% of antibiotics penicillin-streptomycin. Cells were maintained at 37 °C under humidified atmosphere (5% CO_2_ and 95% relative humidity). Cells were cultured in flasks (Corning, EEUU) until reaching 80% confluence and sub-cultured using 0.25% EDTA-trypsin (Thermo Fisher, EEUU) for 5 min. They were recovered by centrifugation at 800 rpm for 10 min. Cells from the third to twelfth passage were seeded at a density of 500,000 cells/well in 6-well plates for western blot experiments.

#### 2.2.16. Western Blot

Cell samples were lysed in ice-cold AKT lysis buffer (137 mM of NaCl, 20 mM of NaF, 10% glycerol, 20 mM of Tris–HCl, 1% Nonidet P-40, 1 μg/mL of leupeptin, 1 mM of phenylmethylsulfonylfluoride, 1 mM of sodium pyrophosphate, and 1 mM of Na_3_VO_4_, pH 7.5). Protein quantification was performed using the Pierce BCA Protein Assay Kit (ThermoFisher, Madrid, Spain) following manufacturer’s instructions. Then, 30 μg of protein were resolved by sodium dodecyl sulfate-polyacrylamide gel electrophoresis (SDS-PAGE) and transferred to Immobilon-P polyvinylidene difluoride (PVDF) membranes (Millipore Corp.). Membranes were activated with methanol and blocked with 4% bovine serum albumin (BSA) in Tris-buffered saline-Tween (TTBS: 10 mM of Tris, 150 mM of NaCl; 0.2% Tween-20, pH 7.4) for 2 h. After that, membranes were incubated overnight at 4 °C with the corresponding primary antibodies: anti–HMOX1 (1:1000, ab68477, Abcam, Cambridge, UK), anti-glutamate-cysteine ligase catalytic subunit (GCLC, 1:1000, ab41463, Abcam), anti-β-actin (1:50,000, A3854, Sigma–Aldrich, Madrid, Spain). Peroxidase-conjugated secondary antibodies (1:10,000, Santa-Cruz Biotechnology, Heidelberg, Germany) were used after TTBS washings for 1 h to detect proteins by enhanced chemiluminescence using the ECL Advance Western-blotting Detection Kit (GE Healthcare, Amersham, The Netherland) and the ChemiDoc MP System (Bio-Rad Laboratories, Madrid, Spain). Band intensities from specific proteins were analyzed with Fiji software.

#### 2.2.17. MTT Method for Cell Viability Measurement

After treatments, primary mixed glial cultures and SH-SY5Y, AREc32, and MEF cells were incubated for 120 min with tetrazolium salt (3-(4,5-dimethylthiazol-2-yl)-2,5-diphenyltetrazolium bromide solution (MTT, 0.5 mg/mL) for cell viability measurement. In this assay, MTT (yellow salt) was reduced to purple insoluble formazan crystals by oxidoreductase enzymes from viable cells. Then, the formazan crystals were solubilized by adding dimethyl sulfoxide (DMSO) to the corresponding p96-well plates. Finally, absorbance was measured at 535 nm in a microplate reader SPECTROstar Nano (BMG Labtech, Ortenberg, Germany). Basal absorbance was set to 100% and results were normalized to basal conditions.

#### 2.2.18. Molecular Docking on MAO-B

Docking was performed with Schrödinger software using Glide [63]. First, ligand states were produced at pH 7.4 using Epik [64]. Afterwards, they were prepared and minimized using Lig-Prep module [65]. PDB-ID structures were prepared and minimized with the Protein Preparation Wizard tool in Maestro using Optimized Potentials for Liquid Simulations 3 (OPLS3) force field [66]. The box for docking calculations was placed to cover the bipartite MAO-B cavity (both entrance and substrate cavities). Best poses were inspected visually and ranked by energy. The glide ligand docking algorithm was used with standard precision as docking method. All images were constructed with PyMOL software [67].

#### 2.2.19. Molecular Dynamics (MD) Simulations

The selected complexes from docking were employed for MD simulations for 300 ns using Amber18 suite [68]. Ligand parameters were obtained using the Antechamber package in Amber18. In brief, partial charges were attributed with the AM1-BCC charge method [69], employing the general AMBER force field (GAFF) atom types [70]. Ff14SB force field was used for protein parameters and the TIP3P water model was used for protein–ligand complex solvation. Sodium and chloride ions were included to mimic physiological concentration of 0.15 M. Afterwards, the system was submitted to 500 steps of the steepest descent algorithm followed by 500 steps of the conjugate gradient algorithm. An initial 100 kcal mol^−1^ · A^−2^ harmonic potential restriction was applied to the complex and it was gradually lowered. Then, the whole system was minimized and it was heated from 0 to 310 K using the Langevin thermostat in the canonical ensemble (NVT) with complex ^2^ harmonic potential restriction of 2 kcal mol^−1^ · A^−2^. Before starting the production run, the system was finally equilibrated at 310 K in the isothermal–isobaric ensemble (NPT) without harmonic limitation. Analysis of the trajectories was performed using the cpptraj module [71] of Amber18 and they were visualized using VMD [72]. Images of MD simulations were constructed using Pymol software [67].

#### 2.2.20. Statistical Analysis

Data are represented as mean ± S.E.M. IC_50_ and LD_50_ values were calculated by non-linear regression analysis of individual dose-response curves. Experimental and control groups were compared using the *t*-test and multiple groups were compared using a one-way analysis of variance test (one-way ANOVA) followed by a Newman–Keuls post hoc test. Statistical significance was set at * *p* < 0.033, ** *p* < 0.002, and *** *p* < 0.001. Data was analyzed using GraphPad Prism 8.0 software.

## 3. Results and Discussion

### 3.1. Synthesis of the 2-(1H-Indol-3-yl)ethan-1-amine Derivatives

**Scheme 1:**
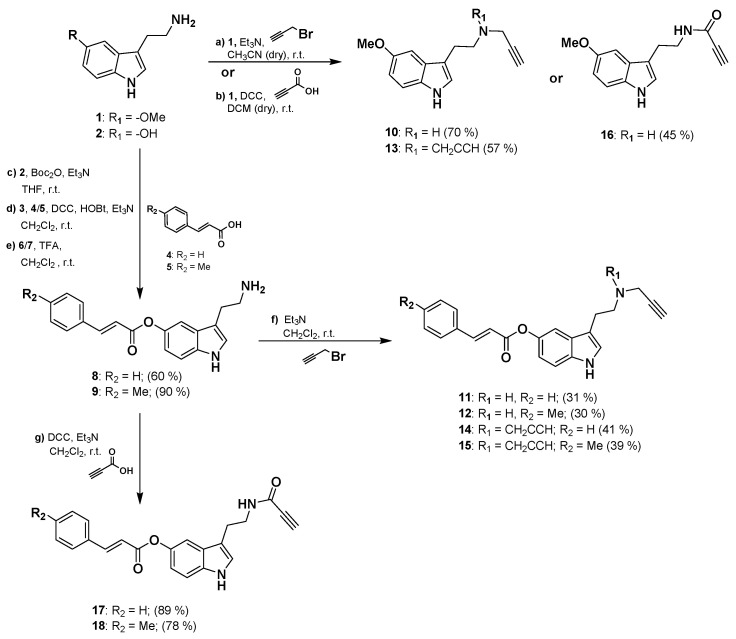
General procedure for the synthesis of **10**–**18** compounds.

Synthesis of the 2-(1*H*-indol-3-yl)ethan-1-amine derivatives **10**–**18** was performed starting from 5-methoxytryptamine, 3-(2-aminoethyl)-1*H*-indol-5-yl-(E)-3-(*p*-tolyl) acrylate or 3-(2-aminoethyl)-1*H*-indol-5-yl cinnamate as shown in Scheme 1. Amine derivatives **10** and **13** were obtained from 5-methoxytryptamine in one reaction by nucleophilic addition to propargyl bromide in presence of Et_3_N. Derivative **16** is readily available by amidation reaction with propiolic acid using DCC as coupling reagent. Amine intermediates **8** and **9** were prepared from the hydroxy-derivative **2** by Boc protection of amine group followed by Steglich type esterification with the corresponding acrylate **4** or **5** in the presence of DCC as a coupling reagent and HOBt as catalyst. Amine intermediates **8**–**9** were finally obtained in high yields by deprotection of the Boc group from **6**–**7** in presence of TFA. Final compounds **11**, **12**, **14**, and **15** were then synthesized with moderate yields in one-step reaction from **8**–**9** by nucleophilic addition to propargyl bromide in presence of Et_3_N. Amide derivatives **17** and **18** were obtained from **8**–**9** by catalytic amidation using the same conditions previously described for **16**. Physicochemical properties predictions for compounds **10**–**18** are shown in Table S1.

### 3.2. Pharmacological Evaluation

#### 3.2.1. NRF2 Induction and MAO Inhibition

As introduced, compounds **10**–**18** were designed as NRF2 inducers. They bear an aryl-acrylate moiety designed to react with key KEAP1 cysteine residues, as previously described by our group [73]. In addition, the different acetylene substitution pattern is expected to modulate this activity based on its electrophilic character. Thus, we first investigated the NRF2 induction potential of our novel derivatives using the stable human mammary MCF7-derived reporter cell line AREc32 [74]. We performed dose–response curves to calculate the corresponding CD values (concentration required to double luciferase expression), summarized in Table 1. In general, novel compounds showed moderate NRF2 induction activity with CD values at the low micromolar range, arising interesting structure activity relationships. As expected, aryl-acrylate moiety inclusion increased NRF2 induction capacity compared to the corresponding -OMe derivatives (**10**, **13**, and **16**). Compounds **11** and **12** (CD = 1.80 ± 0.28 and 2.99 ± 0.26 µM, respectively) showed 7- and 10-fold potentiation compared to **10** (CD = 20.9 ± 2.20); compounds **14** and **15** (CD = 5.07 ± 0.23 and 5.76 ± 0.07 µM, respectively) showed a 3-fold potentiation compared to **13** (CD = 13.9 ± 1.5 µM). These results are in line with the increased electrophilic character of included aryl-acrylate moieties related to their Cys modification mechanism of action to induce NRF2. Considering amine derivatives **10**–**15**, compounds with a single acetylene moiety (i.e., **10**, **11**, and **12**) showed, in general, a slight increase in NRF2 induction activity, possibly due to steric hindrances of double substituted derivatives at regulatory Cys-residues binding sites. Regarding substituents at aryl-acrylate aromatic ring, phenyl derivatives exhibited increased potency compared *p*-tolyl (CD = 1.80 ± 0.28 and 5.07 ± 0.23 µM for compounds **11** and **14**, respectively, compared to CD = 2.99 ± 0.26 and 5.76 ± 0.075 µM for compounds **12** and **15**). These results could be explained by the positive inductive effect of methyl group leading to a reduced electrophilic character. Considering amide derivatives **16**, **17**, and **18**, the propynyl substituent is conjugated with an amide electronic system implementing strong electrophiles resulting in higher potency (e.g., CD = 1.35 ± 0.12 µM for compound **16**). This hypothesis is in line with cellular toxicity observed for these compounds, as potent electrophiles could lead to a glutathione depletion and unwanted toxicity [75] (Supplementary Data; Table S2). Proof thereof is the fact that compounds **17** and **18,** including aryl-acrylate moieties compared to **16**, showed no activity in this assay due to their high toxicity. Importantly, novel compounds demonstrated an improved profile compared to reference compounds melatonin and rasagiline.

Considering MAO-B implication in PD physiopathology and its clinical significance [6], we designed our novel compounds to selectively inhibit this isozyme. Aryl-acrylate moiety inclusion to melatonin scaffold led to novel structures sharing important pharmacophoric features with known inhibitors [76]. Acetylene moieties, which are commonly present in irreversible MAO-B inhibitors, could increase and modulate compounds potency. Therefore, we performed MAO enzymatic assays with dose–response curves to determine the inhibitory capacity and selectivity of compounds **10**–**18**. As shown in Table 1, compounds showed, in general, moderate MAO-B inhibition with IC_50_ values at the micromolar range. Remarkably, all compounds were MAO-B selective inhibitors, except derivative **10** that showed MAO-A selectivity, and compound **16** that was not active. Structure–activity evaluation demonstrates that aryl-acrylate moiety presence is related to higher inhibitory activities (compounds **11** and **12**, IC_50_ = 3.00 ± 0.21 and 3.03 ± 0.27 µM, respectively, compared to **10**, IC_50_ = 77.6 ± 9.1 µM; compounds **14** and **15**, IC_50_ = 17.0 ± 1.6 and 28.1 ± 0.60 µM, respectively, compared to **13**, IC_50_ = 89.1 ± 4.3 µM; compounds **17** and **18**, IC_50_ = 50.0 ± 2.9 and 13.9 ± 1.2 µM, respectively compared to **16**, IC_50_ > 100 µM). This moiety is also related with increased selectivity, these derivatives being at least 2-fold more potent towards MAO-B isozyme compared to MAO-A (compounds **11**, **12**, **14**, **15**, **16**, and **17**). These results might be explained by additional aromatic interactions stablished between this motif and key residues at MAO-B active site.

#### 3.2.2. Antioxidant Activity, Anti-Inflammatory Properties, and Blood–Brain Barrier Permeation Capacity

As introduced, OS plays a central role in PD onset and progression [1,5]. Considering the structural relationship between novel compounds and the free radical scavenger melatonin, it is expected that derivatives **10**–**18** exert a considerable antioxidant activity since melatonin has a direct free radical scavenging effect [77]. Therefore, we used the ORAC assay to test their oxygen derived free radical scavenging capacity. Results are summarized in Table 2. Novel compounds were, in general, more potent scavengers than reference compound Trolox (vitamin E analog). Interestingly, most of them exhibited a similar activity to melatonin being 2-folds more potent than Trolox, except compounds **11** and **12** which showed no scavenging capacity. Considering substituents, we can conclude that compounds bearing a *p*-tolyl substituent were the poorest scavenger of their corresponding subfamily (i.e., compounds **12**, **15**, and **18** from single acetylene, double acetylene and amide derivatives bearing an acetylene moiety, respectively). Importantly, reference compound rasagiline showed no effect in this assay.

Neuroinflammation has been demonstrated to be an essential contributor to PD pathogenesis, being a prominent feature of the disease [79,80]. Regarding the role of NRF2 in controlling several anti-inflammatory proteins [21] and the activation of this target elicited by novel compounds, we next evaluated the potential anti-inflammatory properties of compounds **10**–**18** in LPS stimulated primary mixed glial cultures. LPS binds to toll-like receptors 2 and 4 (TLR2 and TLR4) leading to nuclear factor-κB (NF-κB) signaling activation. NF-κB promotes the expression of different pro-inflammatory genes such as inducible nitric oxide synthase (*iNOS*), NLR family pyrin domain containing 3 (*NLRP3*), and pro-interleukin 1 beta (IL-1β), among others. Mature IL-1β also signals via TLRs to further activate NF-κB [81]. iNOS enzyme over activation increases nitric oxide production and, therefore, nitrite levels. In this model, LPS promotes microglia and astrocytes polarization to a pro-inflammatory state, similarly to disease conditions [82,83]. Novel compounds showed interesting anti-inflammatory properties with EC_50_ values for nitrite reduction ranging from 12.6 ± 0.53 μM of compound **13** to 22.9 ± 3.3 μM of compound **15** (Table 2). Nonetheless, amide derivatives showed significant toxicity in our model, in line with high electrophilic character of this moiety and its potential off-target effects.

To further demonstrate the anti-inflammatory properties of our compounds, we next evaluated the levels of pro-inflammatory cytokine IL-1β in the same model. As shown in Table 2, compounds were able to reduce IL-1β release at 10 μM concentration, exhibiting potencies from 41.1 ± 1.7% of reduction by compound **14** to 68.4 ± 4.9% of reduction by compound **10**. Importantly, there is a cross-talk between NRF2 and NF-κB response pathways, interplaying at several levels. In general, NRF2 pathway activation decreases NF-κB response [84]. Thus, the anti-inflammatory properties of novel derivatives might be related to their NRF2 induction capacity. Similar properties were demonstrated for other NRF2 inducers including sulforaphane, able to reduce cyclooxygenase 2 expression, tumor necrosis factor alpha (TNFα), iNOS, and IL-1β in response to LPS in macrophages [85,86].

Finally, considering their potential use for PD treatment, we explored their blood–brain barrier (BBB) permeability prediction by passive diffusion of compounds **10**–**18** in the PAMPA assay. Results depicted in Table 2 indicate that all compounds are predicted to cross the BBB by passive diffusion with permeability (P_e_) values higher than 4.0 · 10^−6^ cm s^−1^, except compound **12** that showed a low P_e_ value. Compounds **17** and **18** showed uncertain BBB permeability with P_e_ between 4.0 and 2.0 · 10^−6^ cm s^−1^. Results obtained for reference compounds are shown in Table S3 (Supplementary Data).

#### 3.2.3. Neuroprotective Capacity of Compounds **10**–**18** in Oxidative Stress-Related Models

**Figure 1:**
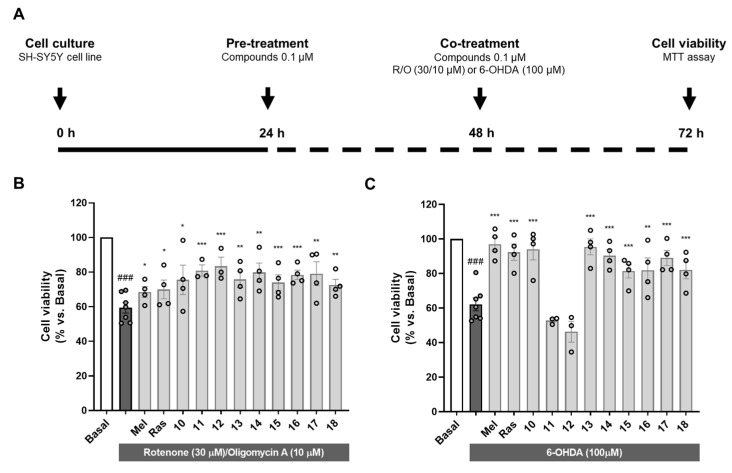
Neuroprotective activity of compounds **10**–**18** against the toxicity exerted by oxidative stress-related toxic stimuli rotenone/oligomycin A mixture and 6-hydroxydopamine. (**A**) Experimental protocol scheme. SH-SY5Y cells were treated with compounds **10**–**18** (0.1 µM) or reference compounds (melatonin and rasagiline, 0.1 µM) during 24 h. Thereafter, cells were treated with compounds **10**–**18**, melatonin or rasagiline, and the corresponding toxic stimuli: (**B**) R/O mixture (30/10 µM) or (**C**) 6-OHDA (100 µM) for 24 h. Cell viability was assessed by the MTT assay. Data are expressed as mean ± SEM of 3–4 independent experiments. Statistical analysis was performed following one-way ANOVA (*p* < 0.05). ### *p* < 0.001 vs. basal condition, * *p* < 0.033, ** *p* < 0.002 and *** *p* < 0.001 vs. the toxic condition after Newman–Keuls post hoc test.

As introduced, OS is one of the main events leading to neurodegeneration in PD [1]. Exacerbated OS status has been widely demonstrated in ex vivo samples of PD patients and associated to mitochondrial dysfunction [87]. Importantly, mutations in antioxidant-related genes such as PD protein 7 (*PARK7*) are associated with autosomal PD and directly correlate with increased cellular OS [88]. Additionally, as previously discussed, nigral dopaminergic neurons are highly vulnerable to OS due to dopamine metabolism by MAO enzymes. Therefore, we selected two in vitro models related to OS to evaluate the neuroprotective potential of compounds **10**–**18** in the human neuroblastoma cell line SH-SY5Y as shown in Figure 1A.

Firstly, we used the rotenone/oligomycin A toxic mixture as a model of mitochondrial dysfunction to induce OS-related toxicity. Rotenone is a mitochondrial complex I inhibitor and oligomycin A is an ATP synthase or complex V inhibitor [89]. This toxic combination impairs mitochondrial respiratory chain and produces a pro-oxidant environment in the cell similar to that observed in PD. Pre- and co-treatment with compounds **10**–**18** at a concentration of 0.1 µM significantly protected cells from the toxic stimulus (Figure 1B). Interestingly, all the compounds showed percentages of neuroprotection higher than the reference compounds melatonin and rasagiline ranging from 32.1% exerted by compound **18** to 53.5% by compound **12** (Supplementary Data; Table S4).

Considering dopamine metabolism as a source of oxidative stress, 6-OHDA is a highly valuable model to reproduce PD pathology. This neurotoxin uses the dopamine transporter to selectively damage dopaminergic neurons. 6-OHDA toxicity is mainly related to its autoxidation leading to exacerbated OS and direct inhibition of mitochondrial respiratory chain complex I, among other events including microglial activation [90]. SH-SY5Y cells possess many characteristics of dopaminergic neurons, expressing tyrosine hydroxylase, dopamine-beta-hydroxylase and dopamine transporter [91]. Similarly to the previous model, pre- and co-treatment with compounds **10**–**18** at a concentration of 0.1 µM led to significant neuroprotection, except for compounds **11** and **12** (Figure 1C). Treatment with the rest of the novel compounds almost completely rescued the cells from the toxic stimulus, with neuroprotection percentages ranging from 41.5% exerted by compound **15** to 87.4% of compound **13** (Supplementary Data; Table S4). Interestingly, the absence of protection observed with compounds **11** and **12** correlates with ORAC results where these compounds were inactive. These results are in line with the strong implication of autoxidation mechanisms in the toxicity elicited by 6-OHDA. In fact, melatonin showed the highest neuroprotection in this model (88.8%). Nevertheless, rasagiline showed a strong protective capacity in this model (77.0%), but no activity in the ORAC assay. This could be explained by its potent MAO inhibition. Like dopamine, 6-OHDA is processed by MAO enzymes, generating ROS [92]; thus, rasagiline could relieve 6-OHDA toxicity by inhibiting MAO-B, but also MAO-A in a medium nanomolar range (IC_50_ = 4.43 nM for MAO-B compared to IC_50_ = 412 nM for MAO-A [93]). This is important considering that MAO metabolism in SH-SY5Y cells is mainly performed by MAO-A isozyme [94].

#### 3.2.4. Compound **14** Upregulates NRF2-Dependet Proteins

After concluding the preliminary pharmacological screening, we aimed to obtain deeper insights into NRF2 activation mechanism.

**Figure 2:**
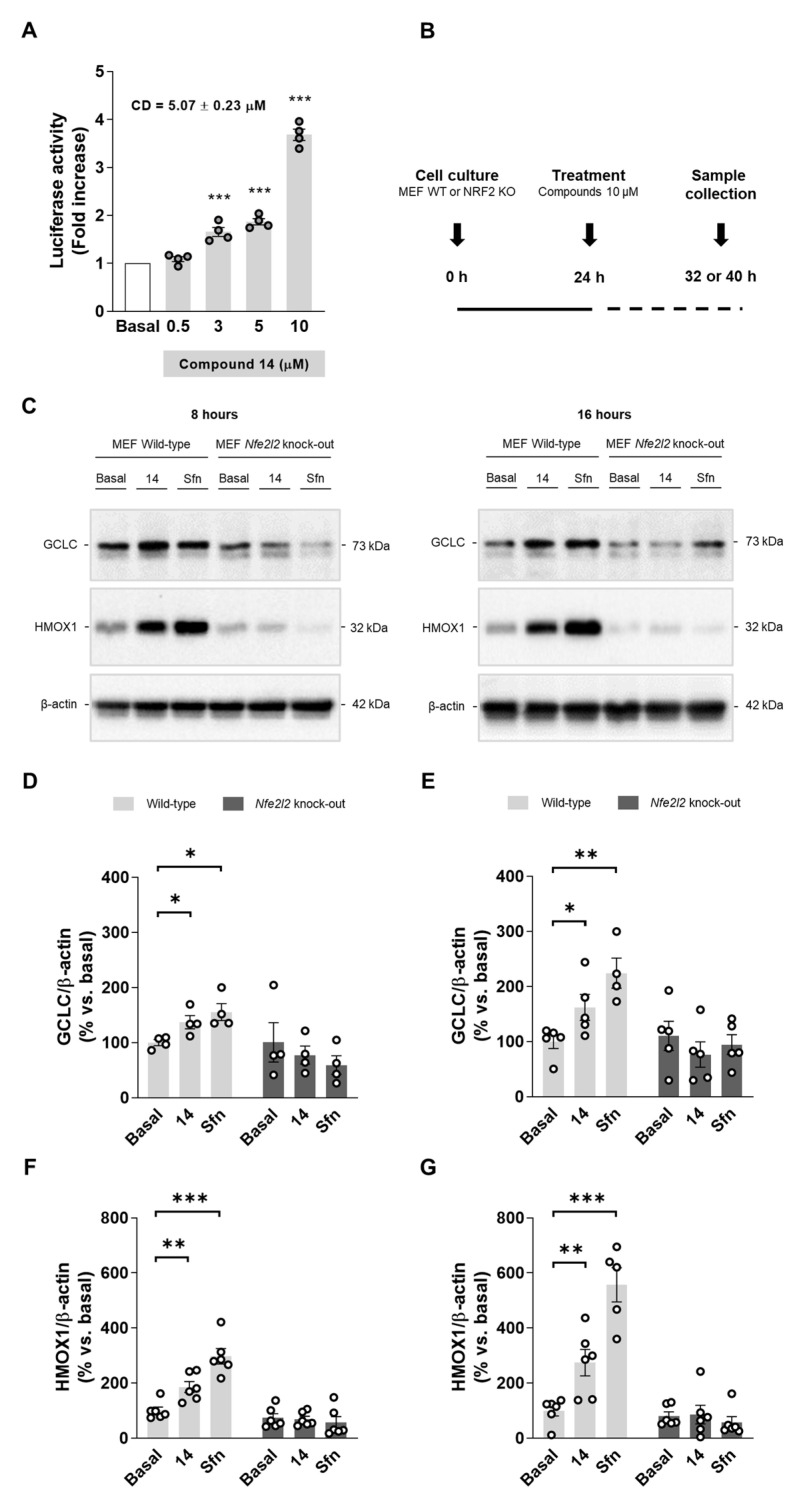
Compound **14** activates the phase II antioxidant response via NRF2 induction. (**A**) Dose–response curve in AREc32 cells treated with compound **14** for 24 h calculated as luciferase activity normalized to basal conditions. (**B**) Experimental protocol followed to evaluate NRF2-related proteins induction. WT or NRF2 KO MEF cells were treated with compound **14** (10 µM) or sulforaphane (10 µM, positive control) across 8 or 16 h. Thereafter, cells were processed for protein levels analysis. (**C**) Representative immunoblots. (**D**,**E**) Quantification of GCLC protein levels for WT and NRF2 KO MEF cells at 8 and 16 h, respectively. (**F**,**G**) Quantification of HMOX1 protein levels for WT and NRF2 KO MEF at 8 and 16 h, respectively. Data are expressed as mean ± SEM of at least 4 independent experiments. Comparisons were made using one-way ANOVA test (*p* < 0.05) followed by the Newman–Keuls post hoc analysis (**A**) or an unpaired *t*-test (**D**–**G**). * *p* < 0. 033, ** *p* < 0.002, and *** *p* < 0.001.

Compound **14** was selected as a hit compound considering its overall pharmacologic profile. Briefly, compound **14** was selected as, among our selective MAO-B inhibitors showing anti-inflammatory properties (i.e., compounds **13**, **14**, and **15**), it was the most potent MAO-B inhibitor and showed potent NRF2 induction capability. Thus, to evaluate its mechanism of action, we measured firstly its capacity to upregulate the phase II antioxidant response. A dose–response curve for NRF2 induction in AREc32 cells is shown in Figure 2A. This result verifies that compound **14** induces NRF2, significantly increasing luciferase activity at 3, 5, and 10 µM. Thereafter, we used WT and NRF2 KO MEF cells to demonstrate a direct activation of the pathway following the protocol detailed in Figure 2B. Upon treatment with compound **14** (10 µM) or sulforaphane (10 µM) as positive control, cells were collected at different times to evaluate different proteins. Successfully, compound **14** increased NRF2-dependent proteins the levels of HMOX1 and GCLC in WT MEF cells (Figure 2C–G). In contrast, compound **14** was not able to increase them in NRF2 KO MEF cells confirming that it exerts its activity through NRF2 activation. Interestingly, HMOX1 and GCLC protein levels increase were time-dependent, as both proteins were further accumulated after 16 h treatment (Figure 2D,F) compared to 8 h (Figure 2E,G). Similar results were observed with the positive control, sulforaphane.

#### 3.2.5. Structural Basis for MAO-B Inhibition

**Figure 3:**
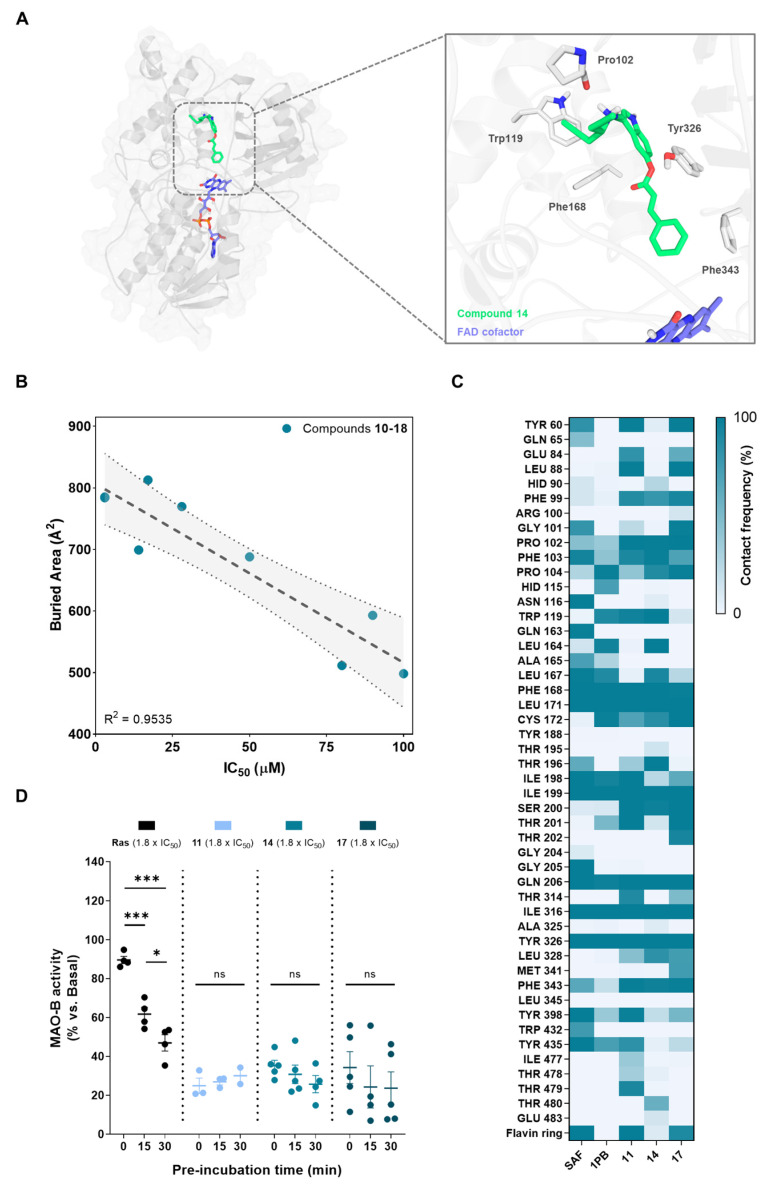
MAO-B binding mode determination. (**A**) Left, global view of the predicted conformation of hit compound 14 in complex with MAO-B after docking and 300 ns molecular dynamics simulation. Right, detailed position at the active site with key protein residues involved in the interaction with the ligand represented as colored sticks. MAO-B protein is represented as gray cartoon and surface. (**B**) Correlation between experimental IC_50_ values for MAO-B inhibition and buried area for protein–ligand interaction. Dotted gray line indicates the linear regression (±95% confidence interval). (**C**) Protein–ligand contact frequency map of safinamide, 1,4-diphenyl-2-butene, and compounds **11**, **14**, and **17** at the active site. Colors represent the percentage of frames in which there is a distance below 5 Å between a certain residue and the ligand. Results were calculated for the 300 ns simulation. (**D**) Reversibility assay for studying MAO-B kinetics. MAO-B activity was measured after different ligand-enzyme pre-incubation times at 1.8 × IC_50_ μM concentration. Data are expressed as mean ± SEM of at least 3 independent experiments. Comparisons were made using one-way ANOVA test (*p* < 0.05) followed by Newman–Keuls post hoc analysis. * *p* < 0. 033 and *** *p* < 0.001.

In order to study the structural mechanisms for MAO-B inhibition of our novel compounds that could lead to further optimization programs, we performed a computational study. For that purpose, we employed docking calculations followed by a 300 ns MD simulation, considering a reversible binding mode based on structural analogy of novel derivatives with other non-covalent inhibitors. We selected a crystal structure of human MAO-B with an active site open conformation (PDB-ID: 2BK3) [95], as our compounds are not expected to fit only in the reactive site cavity close to the FAD cofactor. This is an important assumption since MAO-B isozyme presents two cavities: an entry cavity and a reactive site or substrate cavity, separated by the “gating” residues Ile199 and Tyr326 [96]. In Figure 3A, we show the final and stabilized position of hit compound **14** at MAO-B active site after molecular dynamics simulation. It exhibited a binding mode along the bipartite pocket occupying both entrance and substrate cavities, a result that is frequently observed for large compounds as safinamide [97]. This class of compounds induce an open conformation of Ile199 side chain that, in our case, it is maintained during all MD simulation. Importantly, compound **14** proved to be able to stablish interactions with key enzyme residues. As shown in Figure 3A, it displayed hydrogen bonding with Pro102 residue and π-π stacking interactions with Trp119, Phe168, Tyr326, and Phe343. Interestingly, Tyr326 and Ile199 are described as a key residues for inhibitor recognition and selectivity, as double Ile199Ala/Tyr326Ala mutations led to a protein exhibiting binding properties closer to MAO-A [96]. In that sense, the π-π stacking interaction between the indole core of compound **14** and Tyr326 could be crucial for its activity and selectivity observed. Another significant finding is the fact that compound **14** was shown to locate the phenyl ring towards the aromatic cavity close to the FAD cofactor, interacting with important residues as Phe343, as seen for other potent and selective MAO-B inhibitors recently developed [98]. This aromatic cage is involved in catalysis and substrate specificity [99], thus, this interaction could be highly relevant for its inhibitory potency. In fact, aryl-acrylate moiety inclusion is related experimentally with higher inhibitory activities (Table 1). Similar binding modes were observed for all novel compounds (data not shown).

We then calculated the buried area for compounds **10**–**18**, which measures the size of the interface in the protein–ligand complex. Higher values of this parameter indicate higher interaction surface between tested compound and the protein being considered as a better ligand stabilization at the active site. As represented in Figure 3B, there is a correlation between the buried area and experimental IC_50_ values for MAO-B inhibition. This result indicates that our model could be further used for activity predictions. We also analyzed the contact frequency between ligands and relevant residues at the active site of the enzyme. The resulting contact map is shown in Figure 3C for a representative compound of each subfamily (i.e., compounds **11**, **14**, and **17**) and known inhibitors safinamide and 1,4-diphenyl-2-butene. 1,4-diphenyl-2-butene, which inhibits MAO-B in the same range as our compounds (Ki = 35 µM) [100], did not show relevant additional residue interactions compared to them, again validating the model. Considering safinamide as reference (Ki = 450 nM) [97], we can see slight changes in contacts that could explain the activity variations. As shown in Figure 3C, we can highlight safinamide interactions with Gln65, Asn116, Gln163, Gly205, and Trp432 that are absent in compounds **11**, **14**, and **17**. In contrast, they display some interactions at a specific region of the entrance cavity (Glu84, Leu88, and Phe99) that might be detrimental for their inhibitory capacity, considering that this area is far from the reactive site.

Finally, we performed a reversibility test to evaluate their binding kinetics. This is based on the use of different pre-incubation times of compounds with MAO-B enzyme prior to measure the enzymatic activity. Irreversible inhibitors as rasagiline show decreased activity over time, since they have more time to inhibit the enzyme by covalent bonding. In contrast, MAO-B activity remains time-invariant upon treatment with reversible inhibitors. Results are depicted in Figure 3D, showing a reversible inhibition elicited by representative compounds of each subfamily (i.e., compounds **11**, **14**, and **17**) compared to rasagiline.

#### 3.2.6. Compound **14** Reduces Cell Death and Oxidative Stress Production in Rat Striatal Slices as an Acute Ex Vivo Model of PD

**Figure 4:**
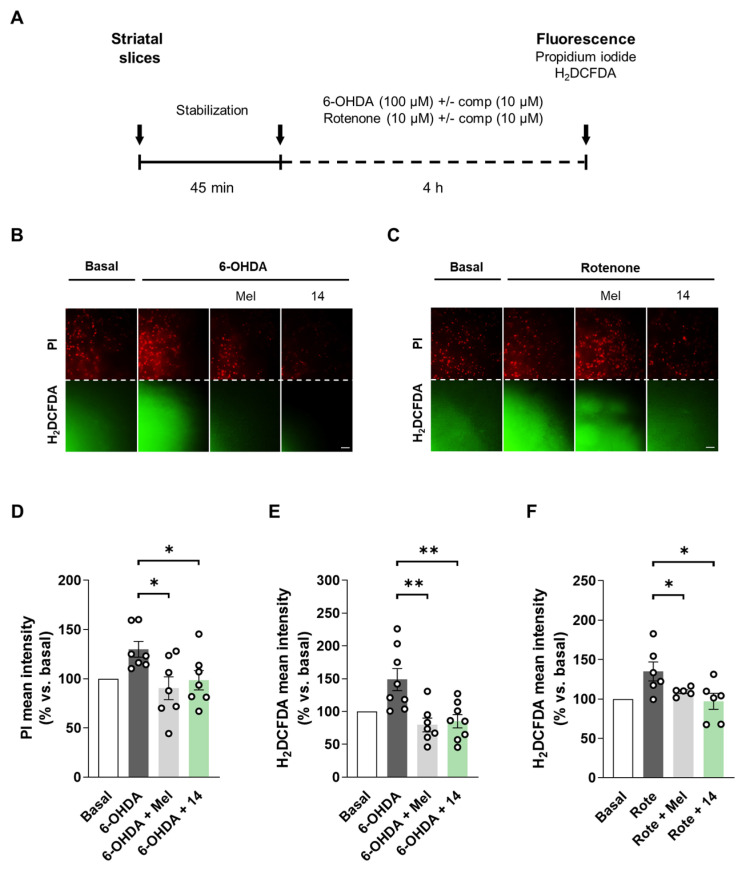
Compound **14** reduced cell death and restored ROS levels in rat striatal slices treated with 6-OHDA or rotenone. (**A**) Experimental protocol scheme. Striatal slices were obtained from adult rats. They were co-treated with compound **14** (10 µM) and 6-OHDA (100 µM) or rotenone (10 µM) for 4 h. Melatonin (10 µM) was included for comparative purposes. (**B**,**C**) Representative fluorescence images for cell death (PI) and ROS production (H_2_DCFDA) for 6-OHDA and rotenone, respectively (Scale bar = 50 µm). Cell death quantification of (**D**) and ROS production (**E**) in the 6-OHDA model. (**F**) ROS production quantification in the rotenone model. Data are expressed as mean ± SEM of at least 5 independent experiments. Comparisons were made using one-way ANOVA test (*p* < 0.05) followed by Newman–Keuls post hoc analysis. * *p* < 0. 033 and ** *p* < 0.002.

Finally, we planned to evaluate the neuroprotective and antioxidant properties of hit compound **14** in adult rat striatal slices treated with rotenone or 6-OHDA. This ex vivo model is considered a more complex experimental setup related to PD. As introduced, PD is characterized by neurodegeneration of the nigrostriatal pathway and striatum constitutes a brain region tightly related with the molecular events occurring during disease progression [1,101]. Previous studies have characterized the 6-OHDA [102,103,104] and rotenone [105] induced toxicity models in striatal slices. Here, we selected adult rat striatal slices subjected to acute 6-OHDA or rotenone toxicity as shown in Figure 4A. After slices dissection and stabilization, they were co-treated with 6-OHDA (100 µM) or rotenone (10 µM) and compound **14** (10 µM) during 4 h. Melatonin (10 µM) was included for comparative purposes. As previously reported, 6-OHDA treatment significantly increased cell death and ROS production [103,104]. Compound **14** treatment led to a significant reduction in cell death measured with the fluorescent dye propidium iodide (Figure 4B,D). Moreover, compound **14** significantly decreased ROS production evaluated with the fluorescent probe H_2_DCFDA (Figure 4B,E). Interestingly, compound **14** had the same protective capacity as melatonin in the 6-OHDA model, completely protecting slices from the toxic stimulus. Previous studies had showed superoxide and nitric oxide increases in striatal slices upon treatment with rotenone [105]. In our model, rotenone treatment also led to an increase in ROS levels, which were reduced by compound **14** or melatonin treatments (Figure 4C,F). Cell death was not significantly altered in this model. Although our compounds were protective in the SH-SY5Y model, striatal slices constitute a more complex scenario. Glia contribution is important in this case, regarding its connection with neurotoxicity related to dopamine metabolism by MAO-B enzyme. Pharmacological profile of compound **14** is relevant in the context of the results obtained, regarding all the biological implications of its main targets.

## 4. Conclusions

The novel 2-(1*H*-indol-3-yl)ethan-1-amine derivatives **10**–**18** were designed as novel NRF2 inducers and selective MAO-B inhibitors for PD treatment. Pharmacological screening of these compounds proved that they were active for their main targets. In that sense, compounds **10**–**18** showed, in general, NRF2 induction activity and selective MAO-B inhibition at a low-to-medium micromolar range. Additionally, they exerted ROS scavenging capacity, similar to their parental compound melatonin, and anti-inflammatory properties in primary mixed glial cultures in terms of nitrites and IL-1β reduction upon LPS stimulation. Compounds **10**–**18** were in general predicted to reach the central nervous system evaluated by PAMPA assay and they were protective in oxidative stress-related models in SH-SY5Y cells. We could stablish structure–activity relationships that explain the potency variations observed in the in vitro experiments. In light of these results, we selected compound **14** as the hit compound based on its overall pharmacological profile to obtain deeper insights into its mechanisms of action. Compound **14** effectively upregulated NRF2-dependent proteins and we confirmed with MEF cells obtained from NRF2 WT and KO mice that this effect was exerted through NRF2 activation. Importantly, this compound reduced cell death and reestablished ROS levels in a more complex model in rat striatal slices treated with 6-OHDA or rotenone. Additionally, computational data allowed us to describe its predicted binding mode at the MAO-B active site, drawing important conclusions for further optimization programs. Our novel approach combining NRF2 induction with selective MAO-B inhibition, exemplified by hit compound 14, could lead future efforts towards the development of a disease-modifying drug candidate for PD treatment.

## 5. Patents

The results here described have been protected in patent application P202230025. Priority date: 14/01/2022.

## Figures and Tables

**Table 1:** Main targets validation for compounds **10**–**18**. NRF2 induction activity in the AREc32 cell line model and MAO inhibition of compounds **10**–**18** and reference compounds. NRF2 induction CD values represent the concentration of compound required to double luciferase activity compared to basal conditions. *Tert*-Butylhydroquinone (TBHQ) was used as positive control of NRF2 induction. MAO inhibition IC_50_ values were calculated from dose–response curves. Data are expressed as mean ± SEM of at least 3 independent experiments.

Compound	CD (µM) NRF2 Induction	IC_50_ MAO-B (µM)	IC_50_ MAO-A (µM)	Selectivity Index (MAO-A/MAO-B)
**Melatonin**	>30	>100	>100	-
**Rasagiline**	NA	0.010 ± 0.0011	>0.1	>10
**TBHQ**	1.82 ± 0.09	NE	NE	NE
**10**	20.9 ± 2.20	77.6 ± 9.10	0.826 ± 0.11	0.0106
**11**	1.80 ± 0.28	3.00 ± 0.21	43.8 ± 6.5	14.6
**12**	2.99 ± 0.26	3.03 ± 0.27	59.1 ± 6.0	19.5
**13**	13.9 ± 1.50	89.1 ± 4.30	>100	>1.12
**14**	5.07 ± 0.23	17.0 ± 1.60	56.6 ± 6.3	3.33
**15**	5.76 ± 0.07	28.1 ± 0.60	>100	>3.56
**16**	1.35 ± 0.12	>100	>100	-
**17**	NA	50.0 ± 2.90	>100	>2.00
**18**	NA	13.9 ± 1.20	81.9 ± 5.4	5.89

NA: not active; NE: not evaluated.

**Table 2:** Antioxidant effect, anti-inflammatory properties, and BBB permeation capacity of compounds **10**–**18** and reference compounds. Free radical scavenging capacity was calculated from dose–response curves evaluating their ability to reduce ROS derived free radicals in the ORAC assay. Nitrite production reduction and IL-1β cytokine levels were evaluated in primary mixed glial cultures stimulated with LPS (1 µg/mL) for 18 h evaluated by the Griess method and ELISA, respectively. EC_50_ values for nitrite reduction were calculated from dose response curves using sulforaphane as positive control. BBB permeability was evaluated by the PAMPA assay. Data are expressed as mean ± SEM of at least 3 independent experiments.

Compound	ORAC (T. eq.)	EC_50_ Nitrite Reduction (μM)	IL-1β Levels % Reduction (10 μM)	PAMPA	
				Pe (10^−6^ cm s^−1^)	Prediction
**Melatonin**	2.83 ± 0.18	25.7 ± 2.5	31.6 ± 7.00	NE	
**Rasagiline**	0.04 ± 0.02	NE	NE	23.5 ± 4.20	CNS +
**Sulforaphane**	NE	1.40 ± 0.30 [78]	NE	NE	NE
**10**	2.47 ± 0.13	16.2 ± 2.50	68.4 ± 4.90	12.3 ± 0.62	CNS +
**11**	0.54 ± 0.07	VR	VR	5.08 ± 1.70	CNS +
**12**	0.13 ± 0.01	VR	VR	1.84 ± 0.48	CNS −
**13**	2.09 ± 0.11	12.6 ± 0.53	67.4 ± 5.80	20.3 ± 7.50	CNS +
**14**	1.98 ± 0.15	19.4 ± 1.40	41.1 ± 1.70	4.15 ± 1.80	CNS +
**15**	1.74 ± 0.10	22.9 ± 3.30	60.5 ± 3.50	4.46 ± 1.90	CNS +
**16**	2.67 ± 0.14	VR	VR	6.64 ± 1.30	CNS +
**17**	2.27 ± 0.08	VR	VR	3.12 ± 1.20	CNS ±
**18**	1.42 ± 0.13	VR	VR	2.04 ± 3.20	CNS ±

NA: not active; NE: not evaluated; VR: high cell viability reduction observed. CNS + (high BBB permeability): Pe (10^−6^ cm s^−1^) > 4.0; CNS—(low BBB permeability): Pe (10^−6^ cm s^−1^) < 2.0; CNS ± (BBB permeability uncertain): Pe (10^−6^ cm s^−1^) between 4.0 and 2.0 [60].

## Data Availability

Data are contained within the article or Supplementary Materials.

## Supplementary Materials

The following are available online at https://www.mdpi.com/article/10.3390/antiox11020247/s1, Table S1: Physicochemical properties; Table S2: Cytotoxicity elicited by compounds **10**–**18** in the SH-SY5Y, AREc32 cell lines and primary glial cells; Table S3: Prediction of the BBB passive permeability of control compounds; Table S4: Neuroprotective activity of compounds **10**–**18**. Original WB images; Figure S1: RMSD calculations of molecular dynamic simulations; copies of NMR spectra.
