# Supplementary material for: Novel Series of Dual NRF2 Inducers and Selective MAO-B Inhibitors for the Treatment of Parkinson’s Disease
Source: Antioxidants (Basel). 2022 Jan 27;11(2):247. doi: 10.3390/antiox11020247 (PMC8868346; doi:10.3390/antiox11020247)
Supplement: Supplementary file 1 [file antioxidants-11-00247-s001.zip › antioxidants-1559842-supplementary.pdf]

Supplementary data

Novel series of dual NRF2 inducers and selective MAO-B inhibitors for the treatment of Parkinson's disease

Pablo Duarte [1,2], Patrycja Michalska [3], Enrique Crisman [1,2,4], Antonio Cuadrado [5] and Rafael León [1,*]

[1] Instituto de Química Médica, Consejo Superior de Investigaciones Científicas (IQM-CSIC), 28006 Madrid, Spain.

[2] Instituto Teófilo Hernando y Departamento de Farmacología y Terapéutica, Facultad de Medicina. Universidad Autónoma de Madrid, 28029 Madrid, Spain.

[3] Chemistry Deparment, Imperial College London, London, UK.

[4] Instituto de Investigación Sanitaria La Princesa (IIS-IS), Hospital Universitario de la Princesa, 28006 Madrid, Spain.

[5] Departamento de Bioquímica, Facultad de Medicina, Centro de Investigación Biomédica en Red Sobre Enfermedades Neurodegenerativas (CIBERNED), Instituto de Investigación Sanitaria La Paz (IdiPaz), Instituto de Investigaciones Biomédicas 'Alberto Sols' UAM-CSIC, Universidad Autónoma de Madrid, Madrid, Spain.

* Correspondence: rafael.leon@iqm.csic.es

TABLE OF CONTENTS

1. Supporting experimental data

1.1. Physicochemical properties 3

1.2. Cytotoxicity. LD_{50} values in several cell cultures employed 4

1.3. PAMPA control compounds (BBB permeability) 4

1.4. Neuroprotection in oxidative stress-related models 5

1.5. Original western blot images 5

1.6. MAO-B binding mode elucidation 10

2. Copies of spectra 11

1. Supporting experimental data

1.1. Physicochemical properties

Physicochemical properties predictions for compounds **10-18** were carried out using SwissADME platform [1]. Compounds did not show any violation of the Lipinski's rule of five. In addition, they were all predicted to be soluble or moderately soluble in water. These results, together with the good permeability values for central nervous system evaluated by PAMPA assay, are important for the drug absorption processes. Furthermore, the parental compound melatonin showed similar physicochemical properties compared to compounds **10-18**, especially closed to those lacking the aryl-acrylate moiety.

Table S1. Physicochemical properties for compounds **10-18**.

Compound	Molecular weight (g/mol)	Log $P_{o/w}$ [a]	Num. H-bond donors	Num. H-bond acceptors	Lipinski [b]	Log S [c]	Solubility class [d]
**Melatonin**	232.28	0.97	2	2	0 violat.	-2.34	Soluble
**10**	228.29	1.68	2	2	0 violat.	-2.36	Soluble
**11**	344.41	3.15	2	3	0 violat.	-4.66	Moderately soluble
**12**	358.43	3.36	2	3	0 violat.	-5.04	Moderately soluble
**13**	266.34	2.35	1	2	0 violat.	-2.79	Soluble
**14**	382.45	3.71	1	3	0 violat.	-5.08	Moderately soluble
**15**	396.48	3.91	1	3	0 violat.	-5.46	Moderately soluble
**16**	242.27	1.15	2	2	0 violat.	-2.84	Soluble
**17**	358.39	2.65	2	3	0 violat.	-5.13	Moderately soluble
**18**	372.42	2.87	2	3	0 violat.	-5-51	Moderately soluble

a) MLOGP: Topological method implemented from Moriguchi I. et al. [2, 3] and Lipinski PA. et al. [4]; b) Lipinski (Pfizer) filter implemented from Lipinski CA. et al. [4]. Violations: more than 5 H-bond donors, 10 H-bond acceptors, the molecular weight is greater than 500 and the calculated MlogP > 4.15; c) Ali: Topological method implemented from Ali J. et al. [5]; d) Solubility class is defined as follows: Log S scale - Insoluble < -10 < Poorly < -6 < Moderately < -4 < Soluble < -2 Very < 0 < Highly.

1.2. Cytotoxicity. LD_{50} values in several cell cultures employed

Table S2. Cytotoxicity elicited by **10-18** compounds in SH-SY5Y, AREc32 and mixed primary glial cultures. Viability was measured as MTT reduction in presence of increasing concentrations of derivatives. LD_{50} values were calculated from dose-response curves. Data are expressed as mean ± SEM of at least 3 independent experiments.

Compound	LD_{50} (μM) SH-SY5Y	LD_{50} (μM) AREc32	LD_{50} (μM) Mixed primary glial cultures
**Melatonin**	> 100	> 30	> 30
**Rasagiline**	> 100	> 30	NE
**10**	> 100	> 30	> 30
**11**	16.4 ± 2.32	7.83 ± 1.2	17.8 ± 2.9
**12**	11.6 ± 0.90	11.1 ± 2.0	7.42 ± 1.3
**13**	> 100	> 30	> 30
**14**	66.5 ± 9.8	> 30	> 30
**15**	48.3 ± 6.6	> 30	> 30
**16**	4.87 ± 0.60	3.83 ± 0.68	8.81 ± 2.2
**17**	9.06 ± 0.31	< 1	0.543 ± 0.14
**18**	6.87 ± 1.3	< 1	1.21 ± 0.24

NE: not evaluated.

1.3. PAMPA control compounds (BBB permeability)

Table S3. Prediction of the BBB passive permeability of control compounds expressed as Pe ± SEM.

Compound	PAMPA	
	Pe (10^{-6} cm s^{-1})	Prediction
**Verapamil**	17.5 ± 4.2	CNS +
**Caffeine**	1.65 ± 1.5	CNS -

1.4. Neuroprotection in oxidative stress-related models

Table S4. Neuroprotective activity of compounds **10-18** against the toxicity exerted by rotenone/oligomycin A mixture (30/10 μM) and 6-hydroxydopamine (100 μM). SH-SY5Y cells were treated with compounds **10-18** (0.1 μM) or reference compounds (melatonin and rasagiline, 0.1 μM) during 24 h. Thereafter, cells were treated with compounds **10-18**, melatonin or rasagiline (0.1 μM) and the corresponding toxic stimuli for 24 h. Cell viability was assessed by the MTT assay. Data are expressed as mean ± SEM of at least 3 independent experiments (N= 3-4 for each experiment). Statistical analysis was performed following one-way ANOVA (p<0.05). *p<0. 33, **p<0.002 and *** p<0.001 *vs* the toxic condition after Newman-Keuls post-hoc test.

Compound	R/O (30/10 μM)		6-OHDA	
	**Cell viability (%)**	**Protection (%)**	**Cell viability (%)**	**Protection (%)**
**Basal**	100		100	
**Toxic**	59.4 ± 3.3		62.2 ± 4.0	
**Melatonin**	70.0 ± 6.3	22.3*	97.0 ± 4.7	88.8***
**Rasagiline**	68.5 ± 3.7	28.9*	92.4 ± 5.4	77.0***
**10**	75.6 ± 9.9	44.6*	94.0 ± 7.1	83.5***
**11**	80.9 ± 4.0	48.6***	53.2 ± 1.6	NA
**12**	83.4 ± 6.4	53.5***	46.1 ± 7.2	NA
**13**	75.8 ± 5.5	42.7**	95.4 ± 5.3	87.4***
**14**	79.8 ± 6.2	53.3***	90.3 ± 4.2	71.7***
**15**	74.0 ± 5.2	36.4**	81.4 ± 4.6	41.5***
**16**	78.5 ± 3.2	47.3***	81.9 ± 8.3	48.1**
**17**	79.1 ± 7.9	49.3**	89.0 ± 5.0	58.4***
**18**	72.5 ± 3.9	32.1**	82.1 ± 6.0	43.8***

NA: not active

1.5. Original western blot images

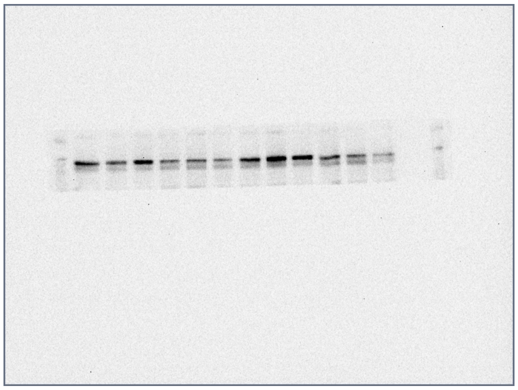

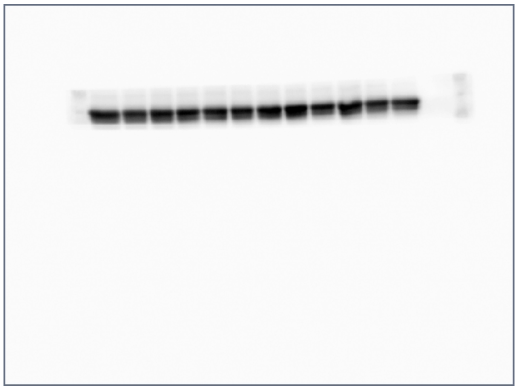

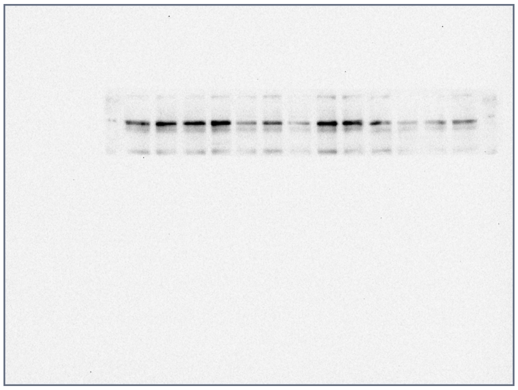
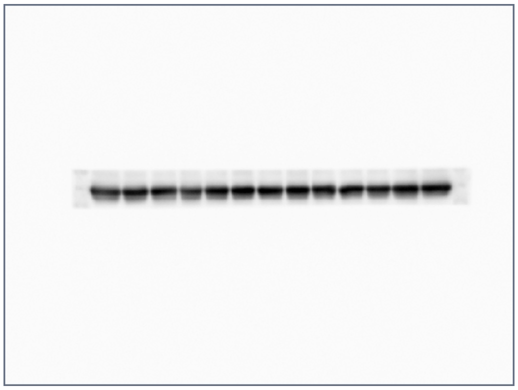
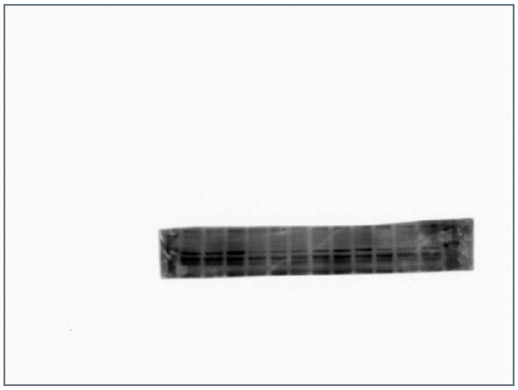
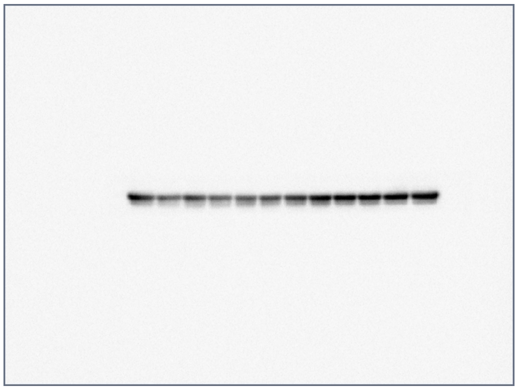
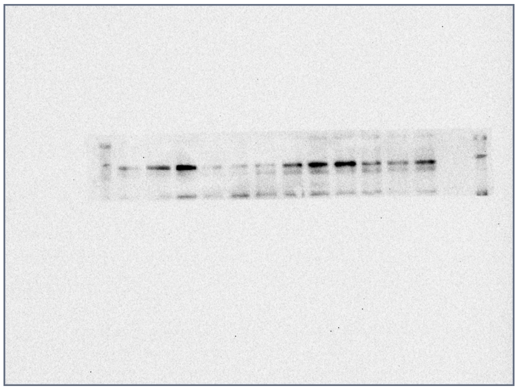
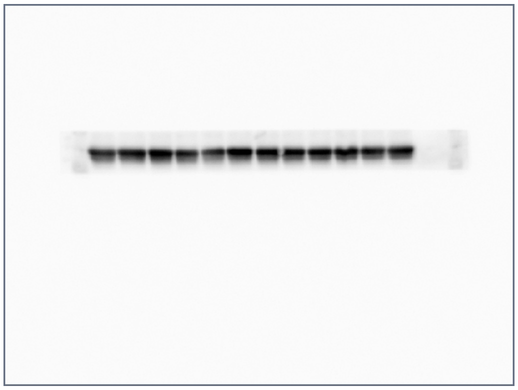

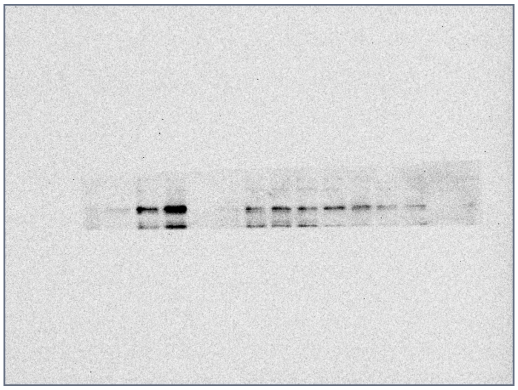
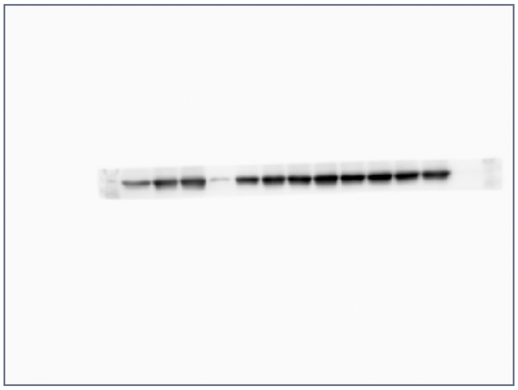
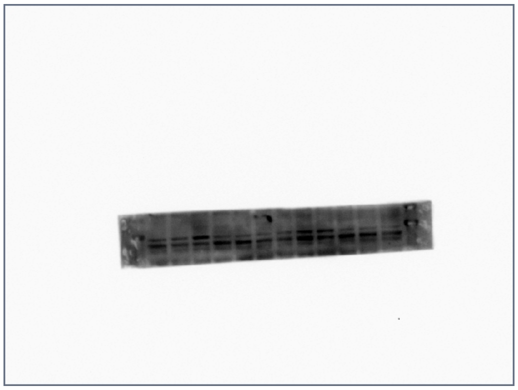
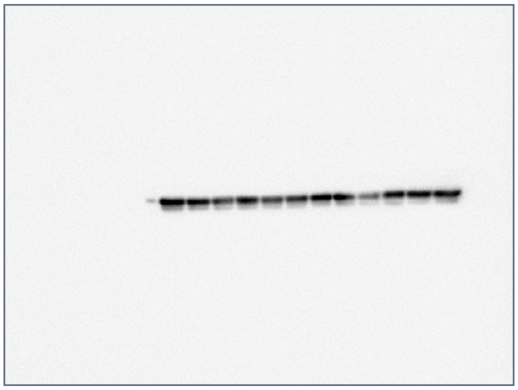
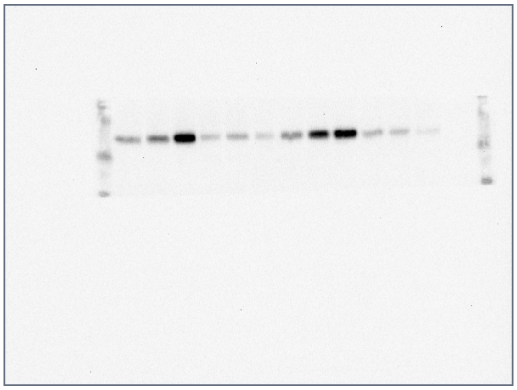
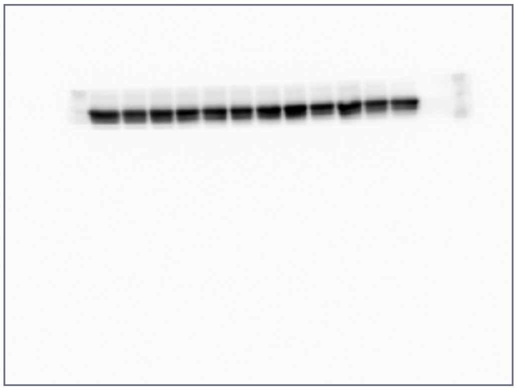

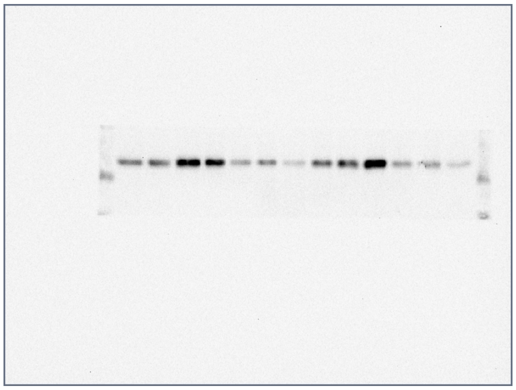
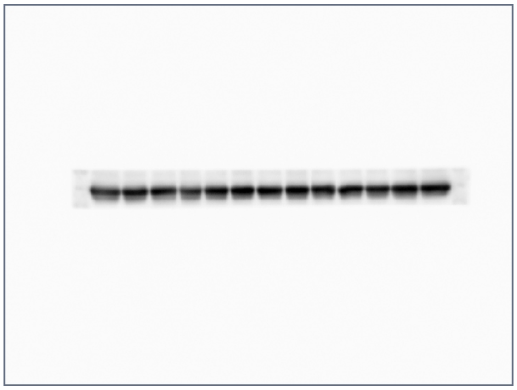
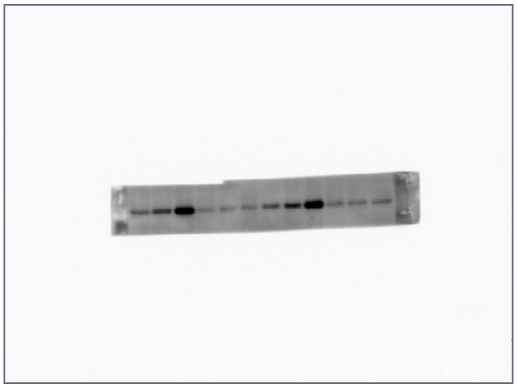
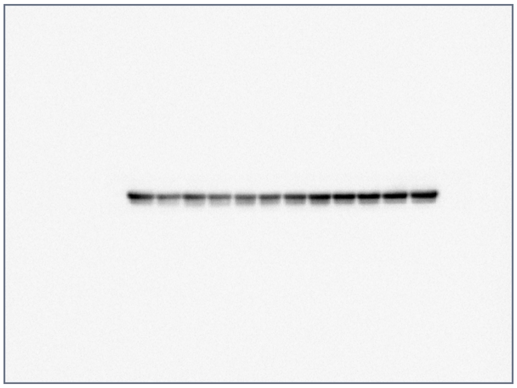
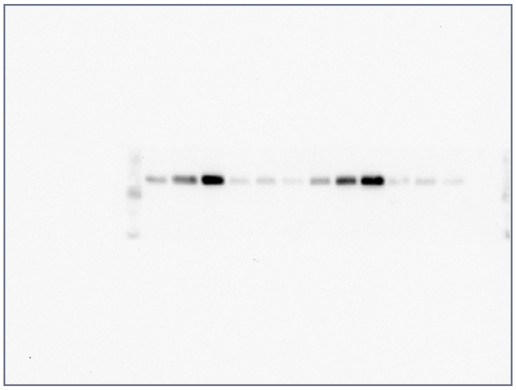
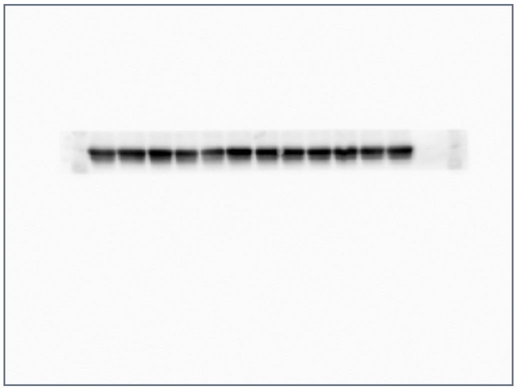

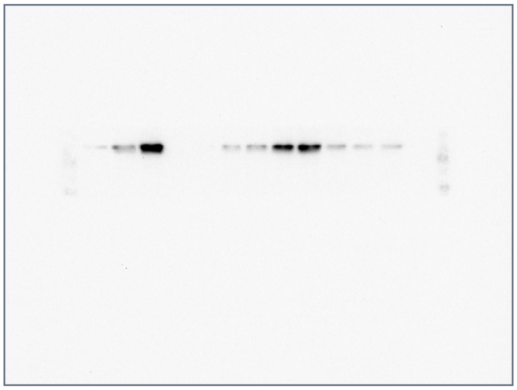

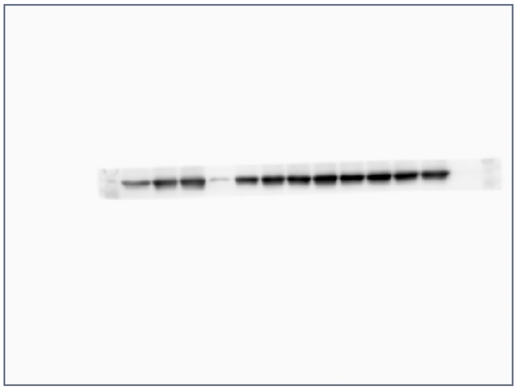

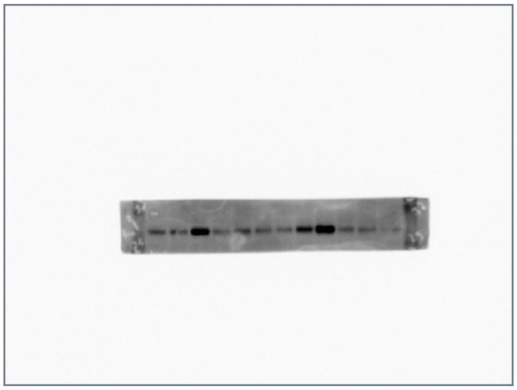

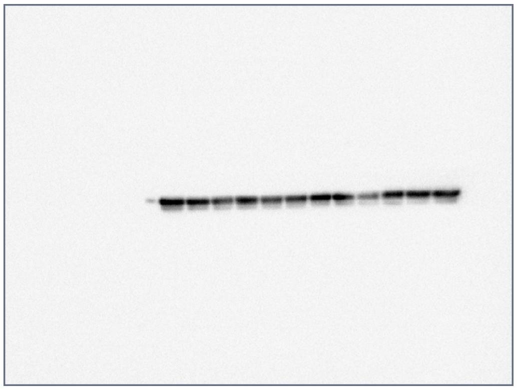

1.6. MAO-B binding mode elucidation

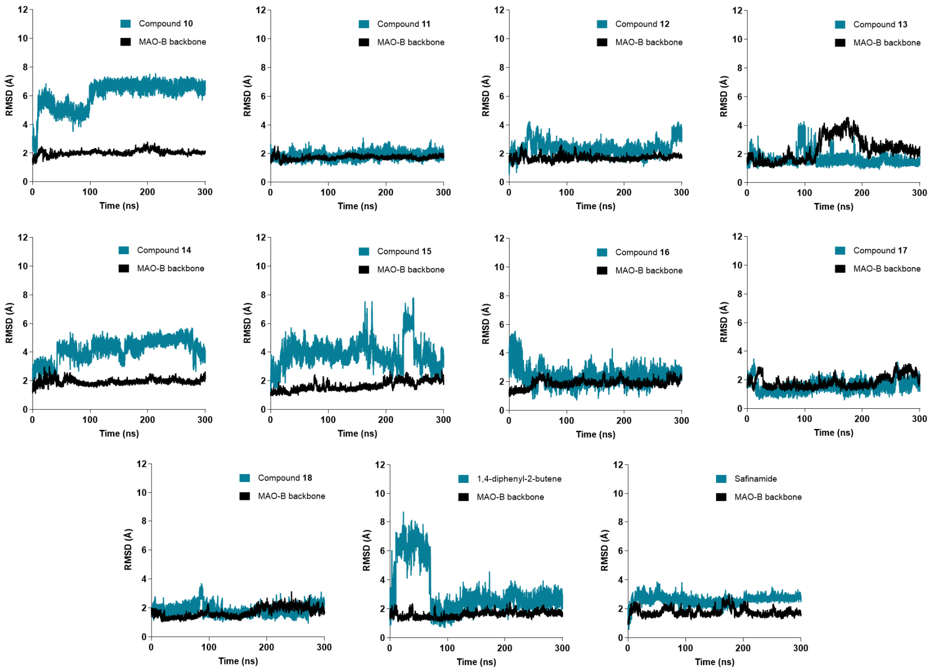

Figure S1. Root-mean-square deviation of atomic positions (RMSD) calculations during molecular dynamics simulations. RMSD is calculated for MAO-B backbone, compounds **10-18**, and reference compounds safinamide and 1,4-diphenyl-2-butene.

3. Copies of spectra

3-(2-((*tert*-butoxycarbonyl)amino)ethyl)-1*H*-indol-5-yl cinnamate (6)

^{1}H NMR (300 MHz, DMSO)

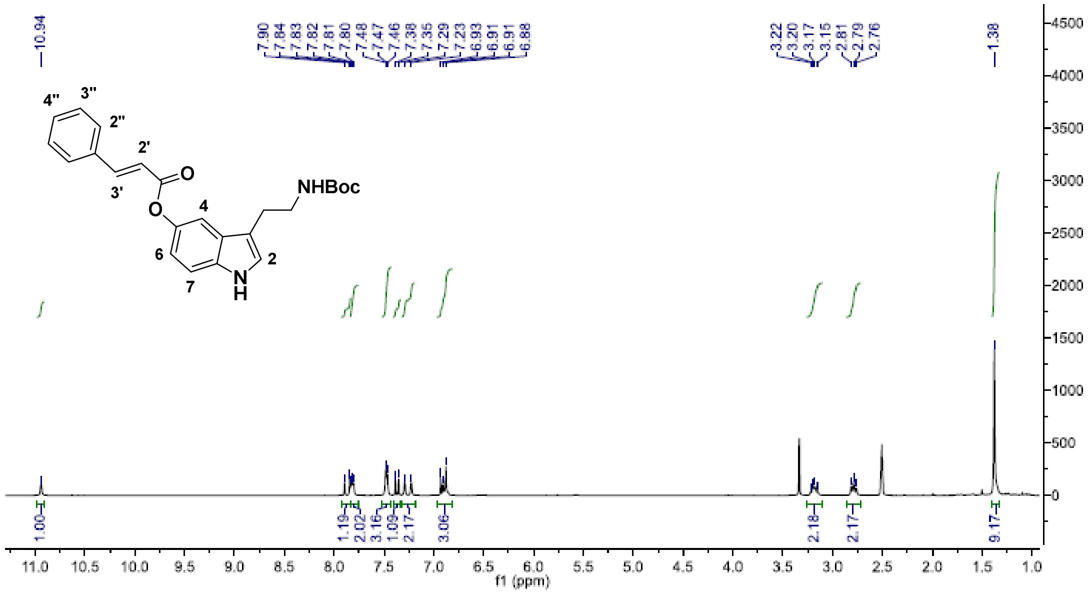

^{13}C NMR (75 MHz, DMSO)

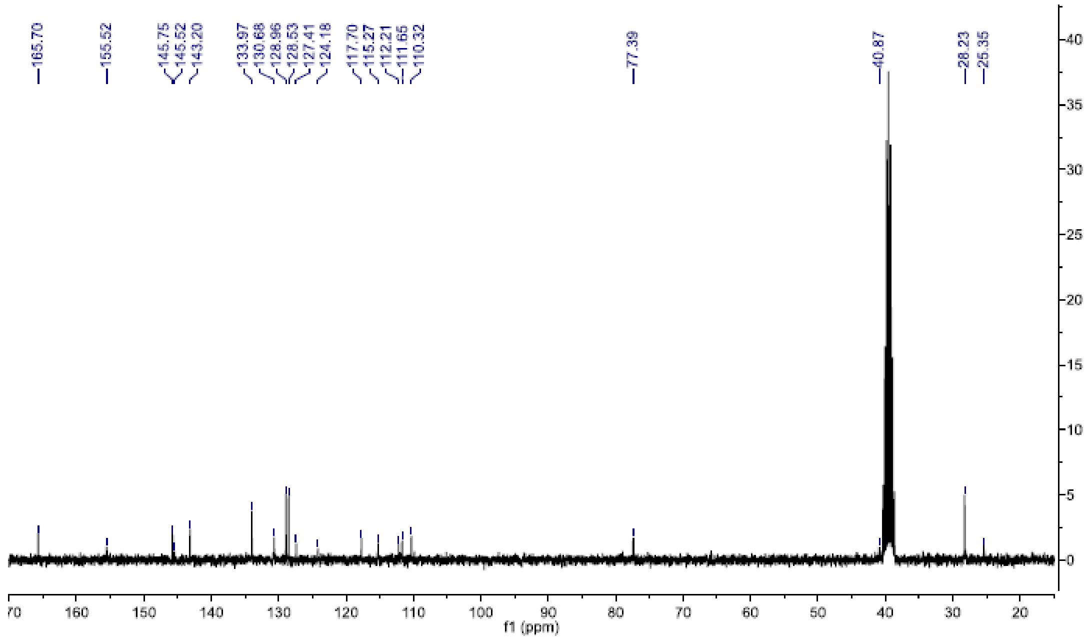

(*E*)-3-(2-((*tert*-butoxycarbonyl)amino)ethyl)-1*H*-indol-5-yl 3-*p*-tolylacrylate (7)

^{1}H NMR (300 MHz, DMSO)

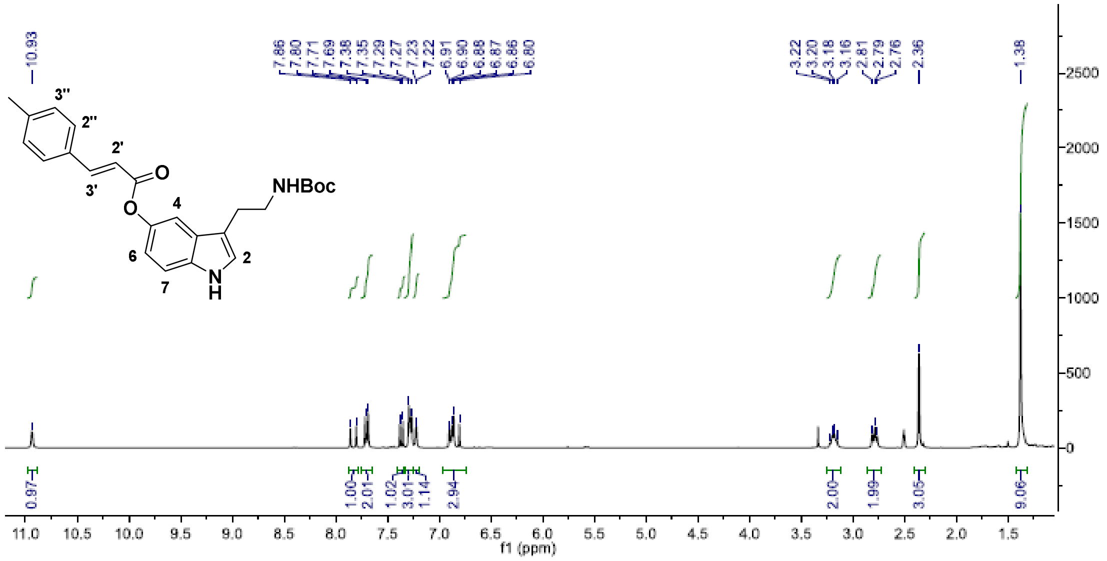

^{13}C NMR (75 MHz, DMSO)

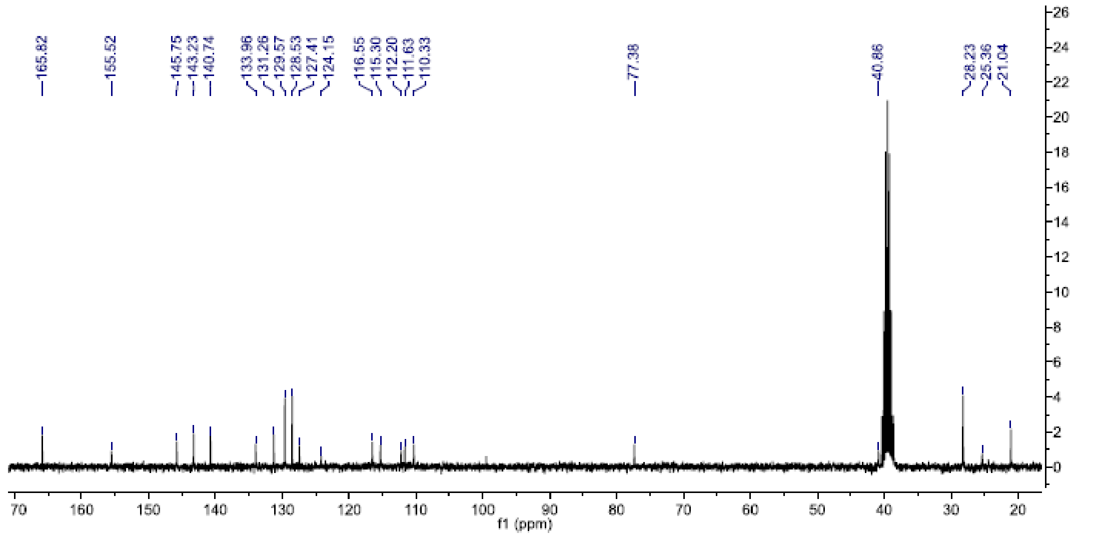

3-(2-aminoethyl)-1*H*-indol-5-yl cinnamate (8)

^{1}H NMR (300 MHz, DMSO)

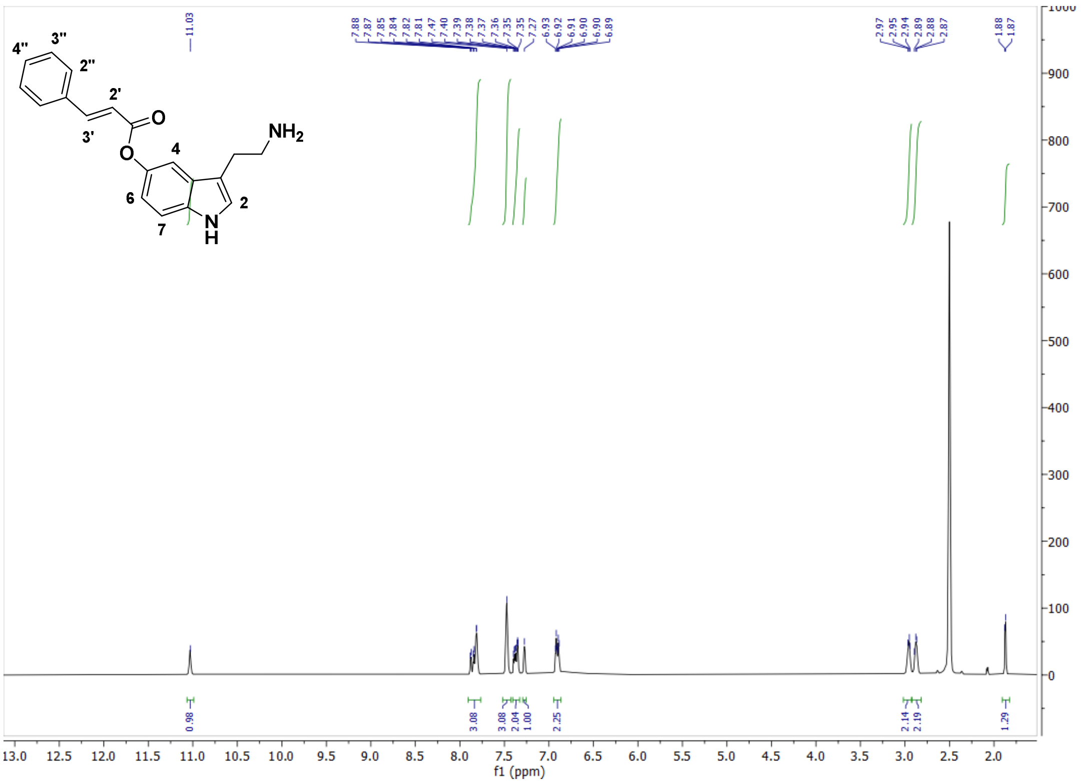

^{13}C NMR (75 MHz, DMSO)

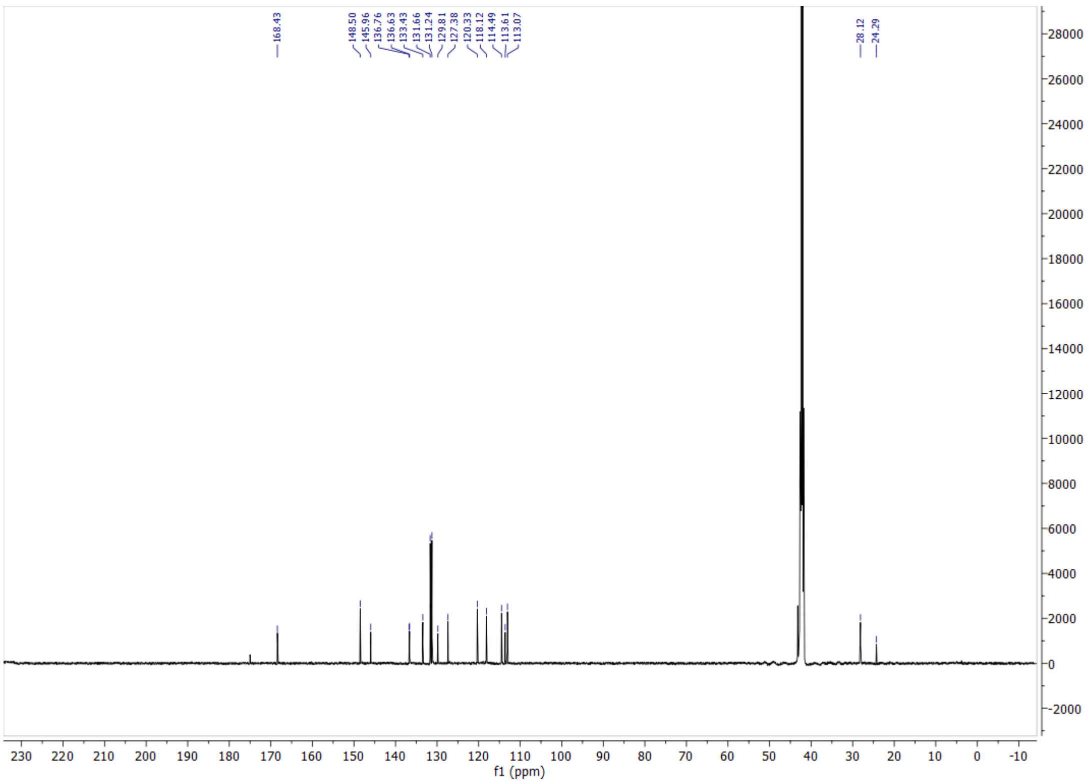

3-(2-aminoethyl)-1*H*-indol-5-yl (*E*)-3-(*p*-tolyl)acrylate (9)

^{1}H NMR (300 MHz, DMSO)

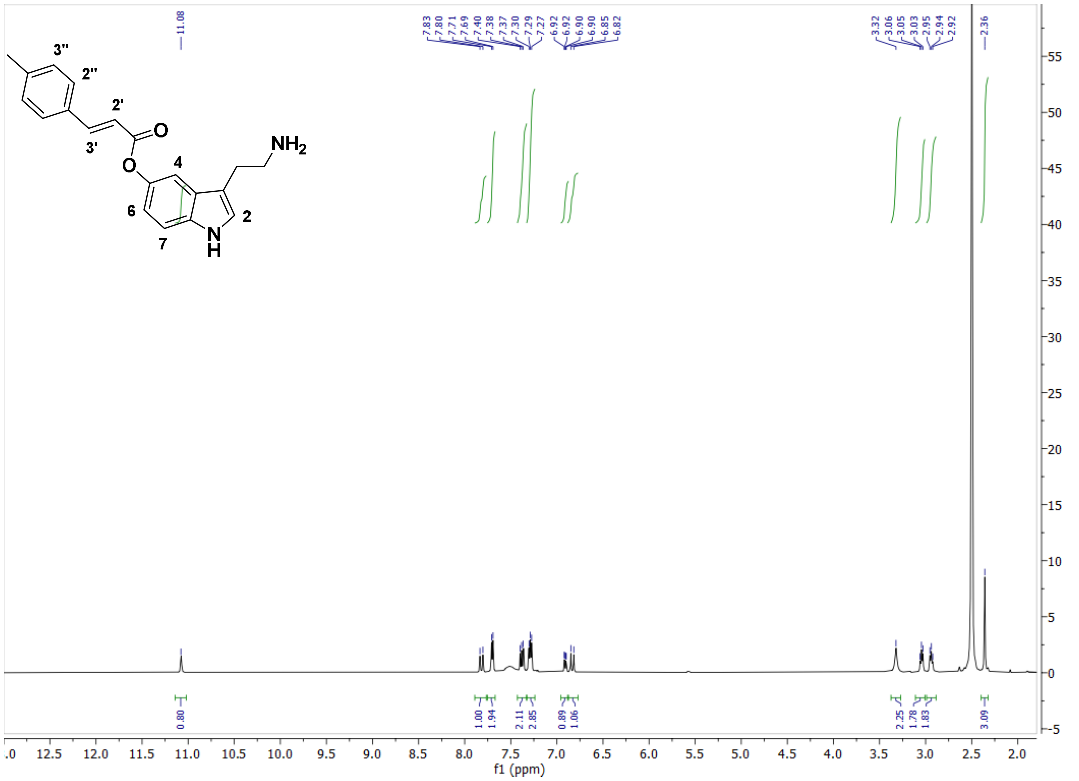

^{13}C NMR (75 MHz, DMSO)

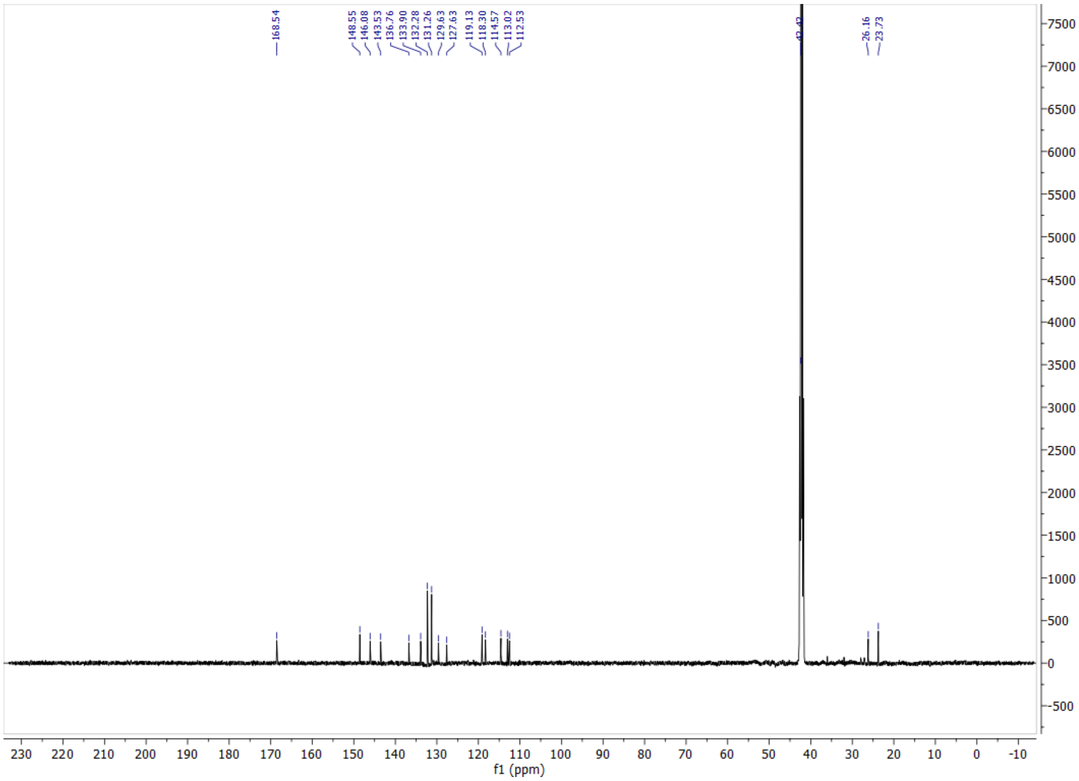

N-(2-(5-methoxy-1*H*-indol-3-yl)ethyl)prop-2-yn-1-amine (10)

^{1}H NMR (300 MHz, DMSO)

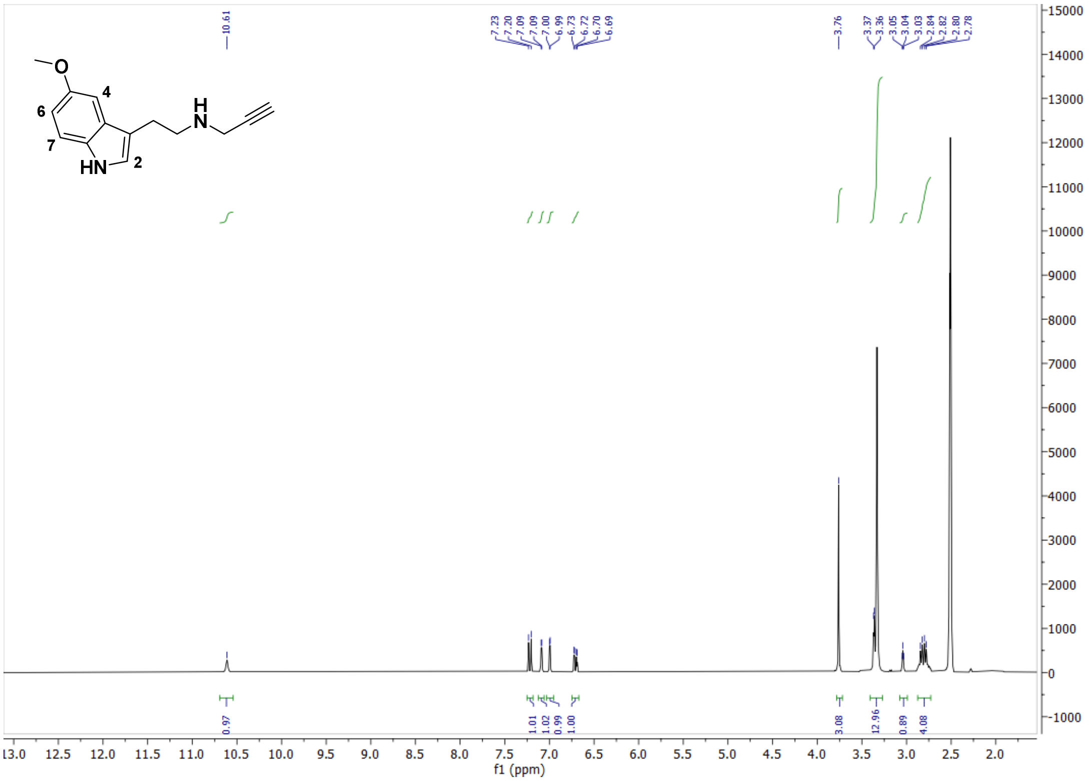

^{13}C NMR (75 MHz, DMSO)

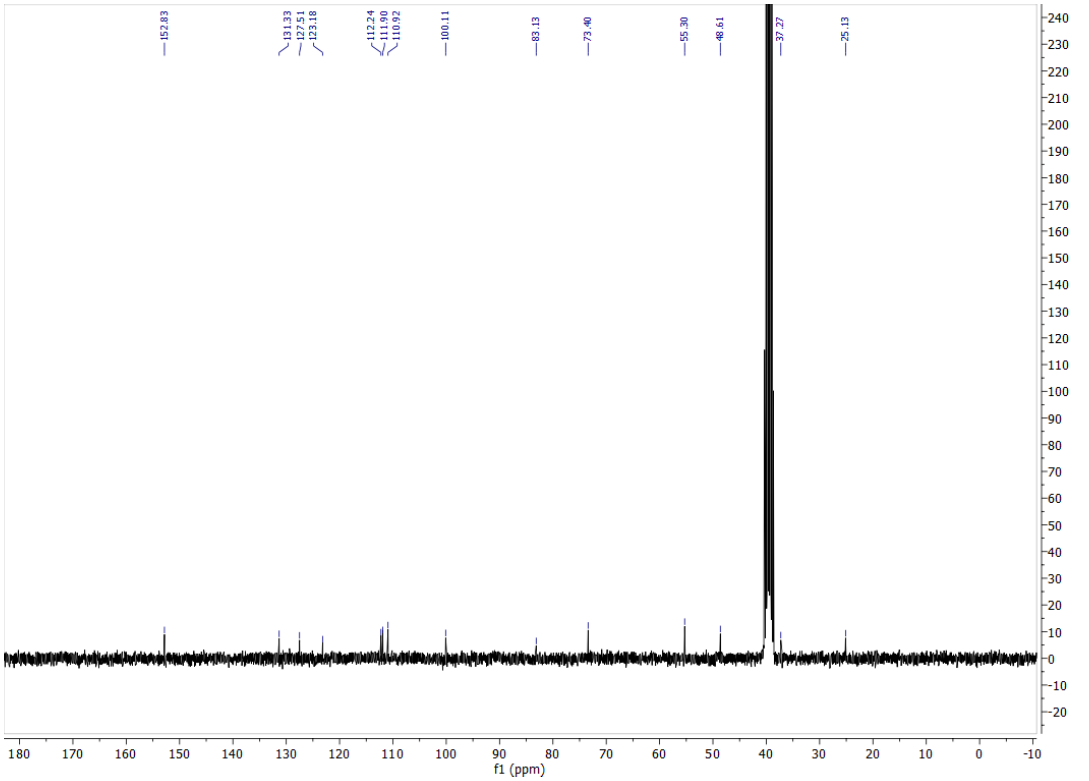

3-(2-(prop-2-yn-1-ylamino)ethyl)-1*H*-indol-5-yl cinnamate (11)

^{1}H NMR (300 MHz, DMSO)

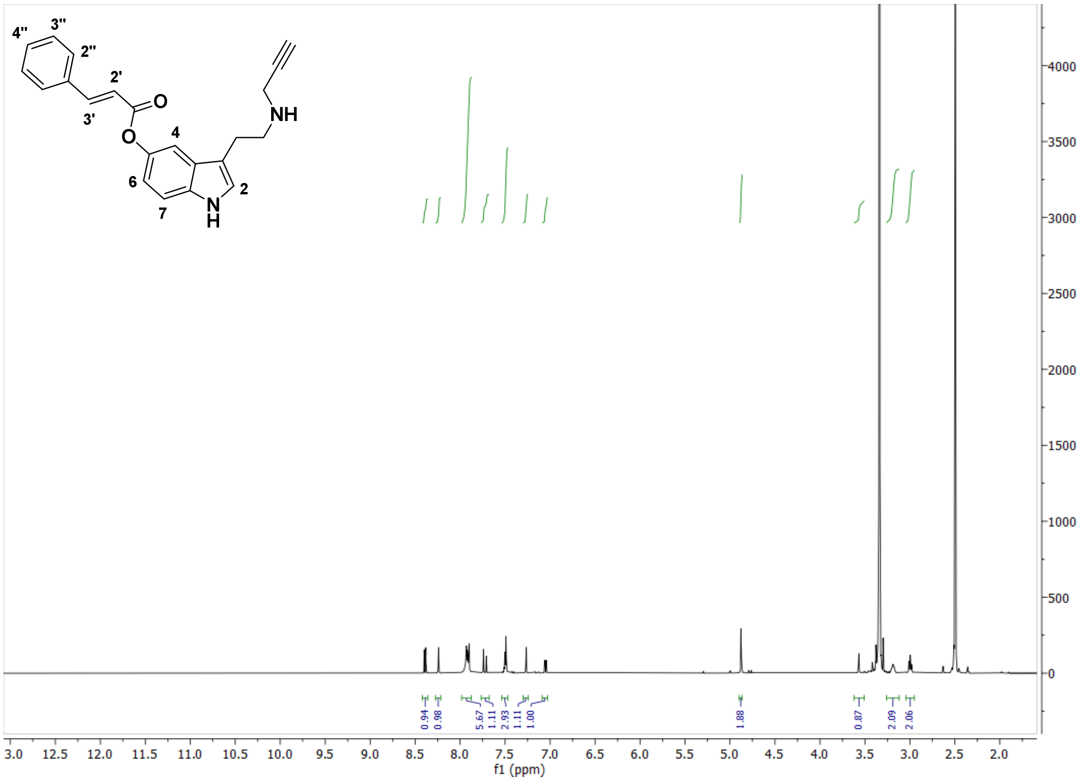

^{13}C NMR (75 MHz, DMSO)

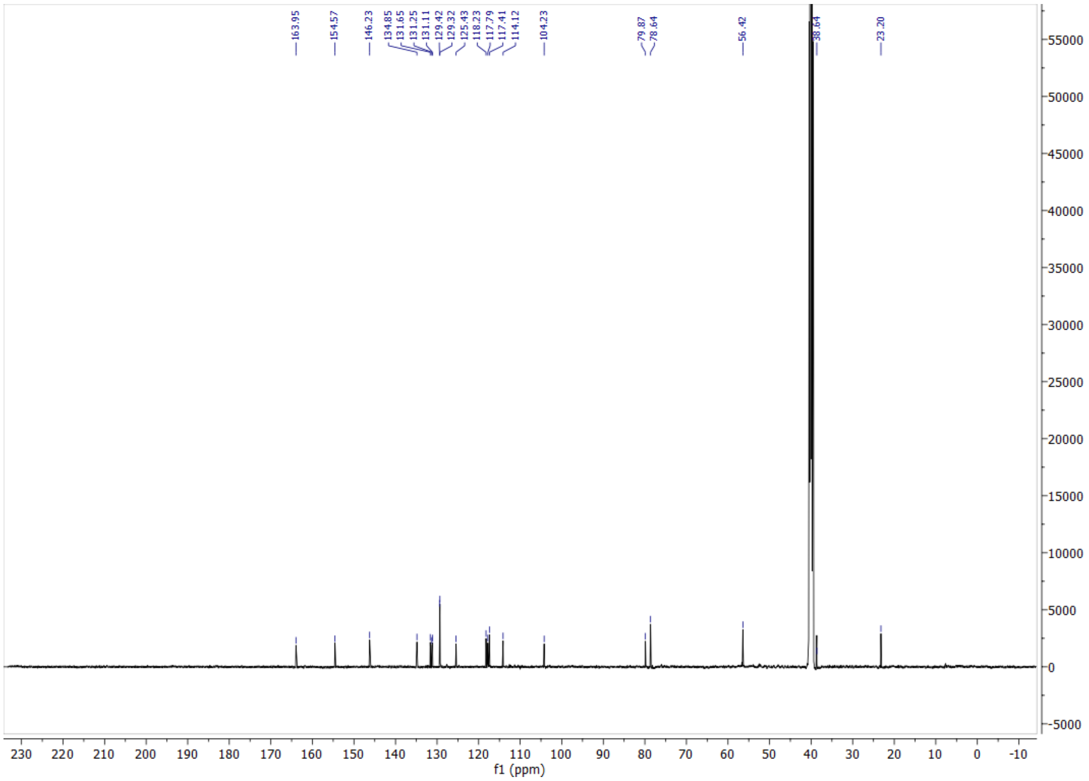

3-(2-(prop-2-yn-1-ylamino)ethyl)-1*H*-indol-5-yl (*E*)-3-(*p*-tolyl)acrylate (12)

^{1}H NMR (300 MHz, DMSO)

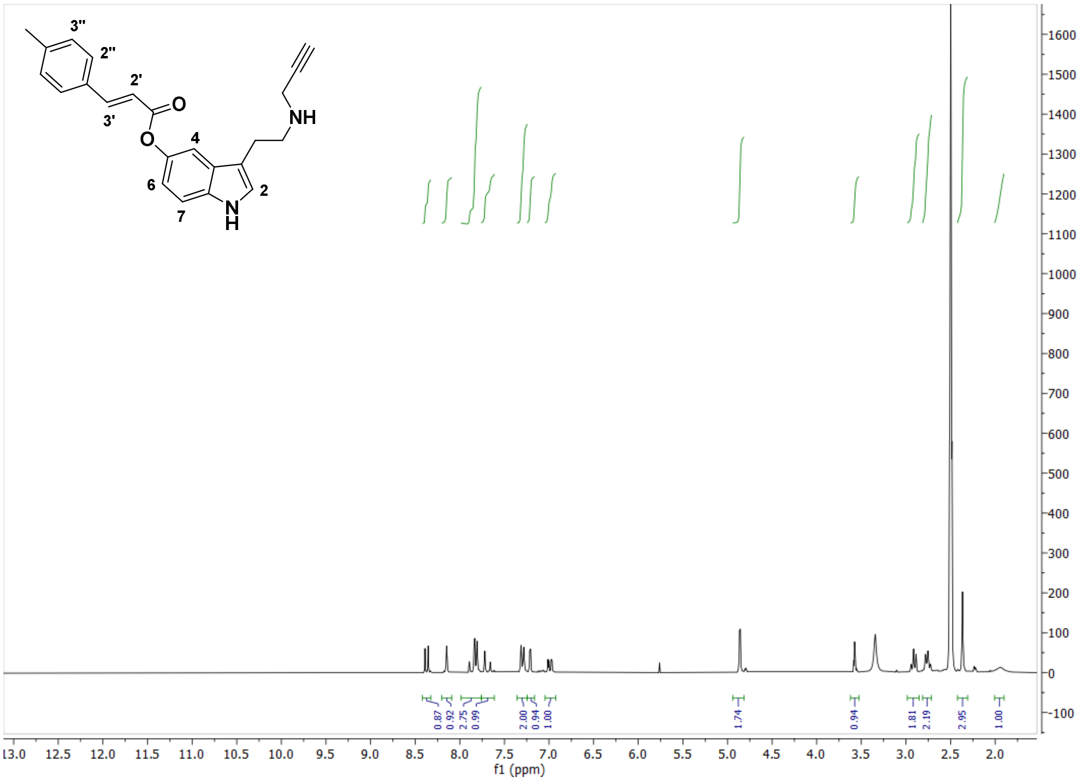

^{13}C NMR (75 MHz, DMSO)

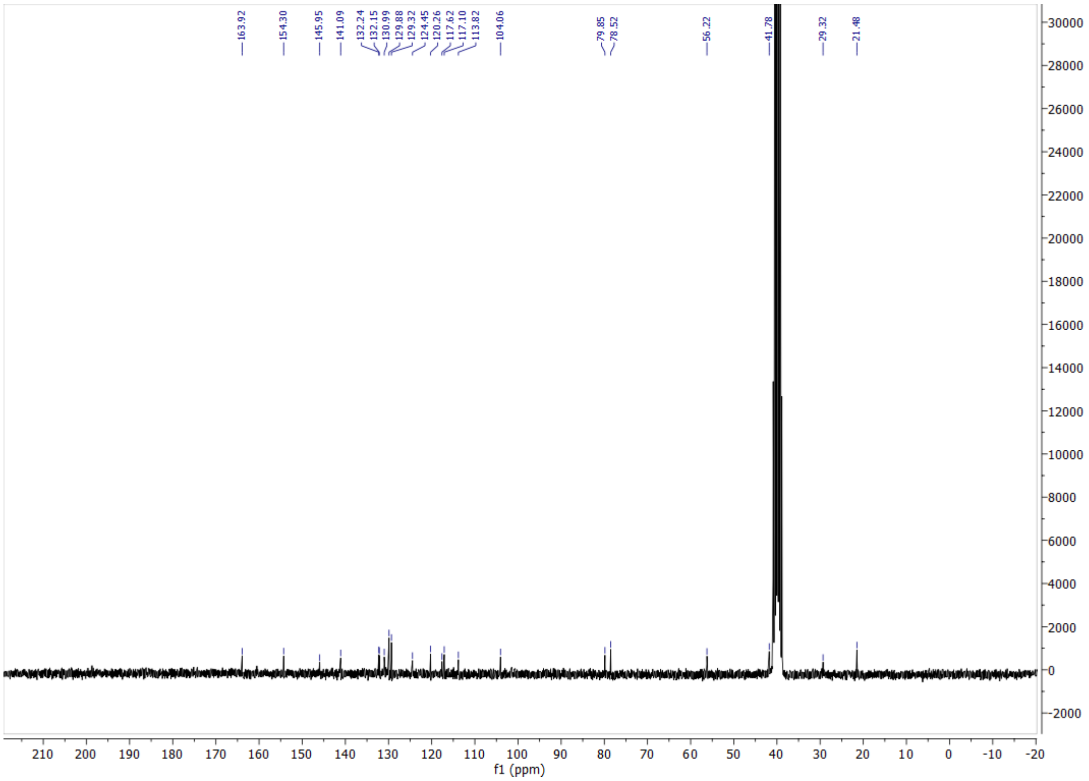

N-(2-(5-methoxy-1*H*-indol-3-yl)ethyl)-*N*-(prop-2-yn-1-yl)prop-2-yn-1-amine (13)

^{1}H NMR (300 MHz, DMSO)

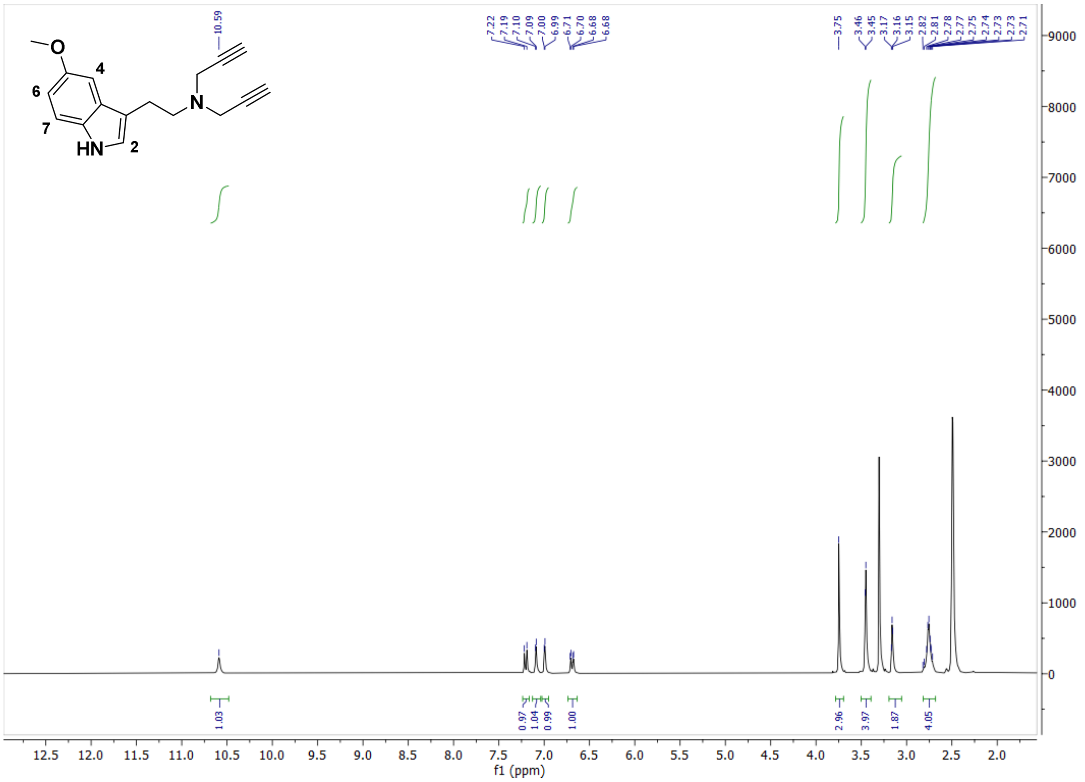

^{13}C NMR (75 MHz, DMSO)

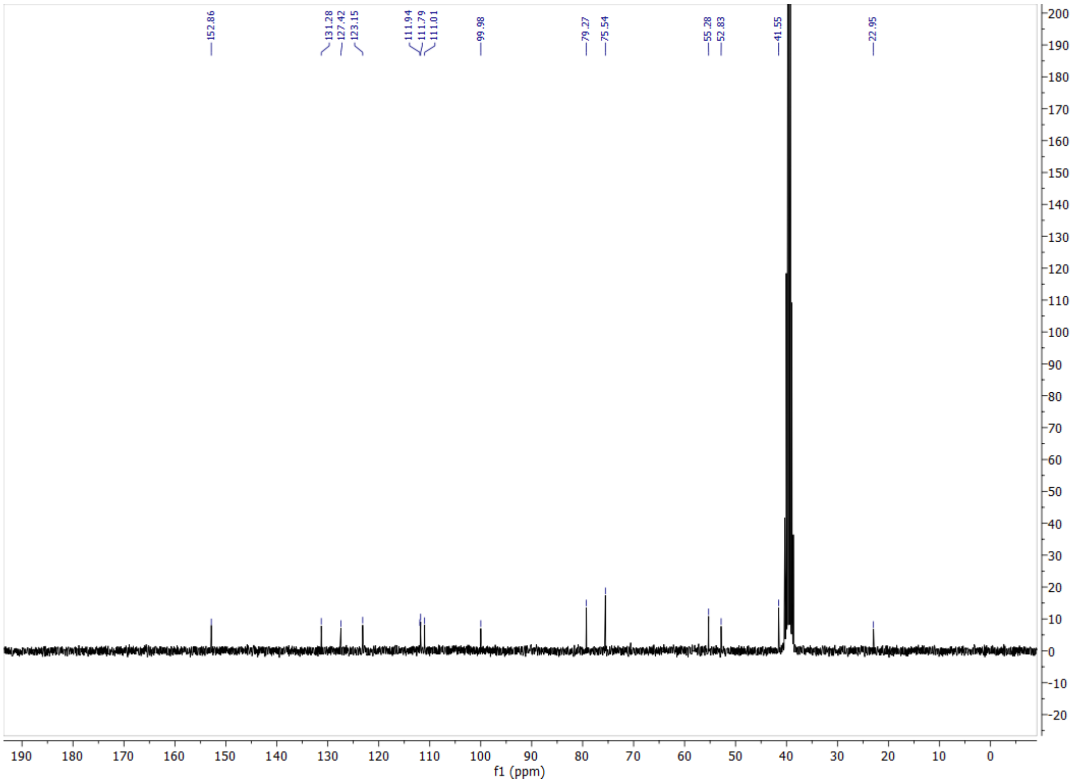

3-(2-(di(prop-2-yn-1-yl)amino)ethyl)-1*H*-indol-5-yl cinnamate (14)

^{1}H NMR (300 MHz, DMSO)

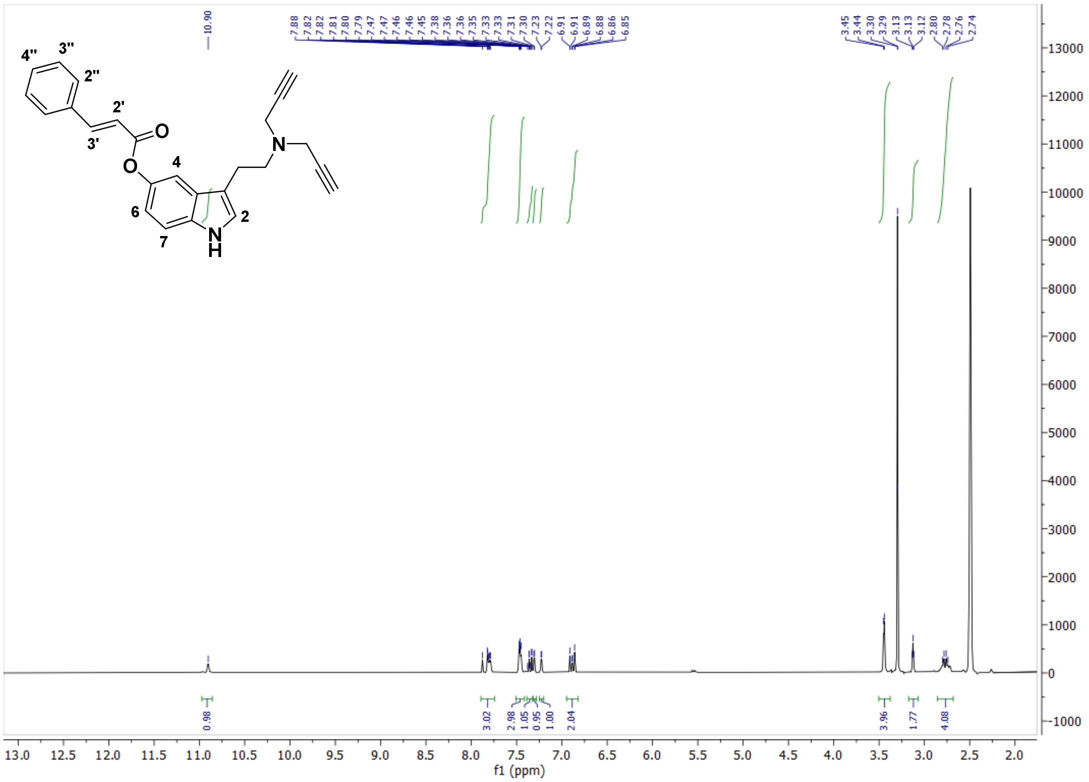

^{13}C NMR (75 MHz, DMSO)

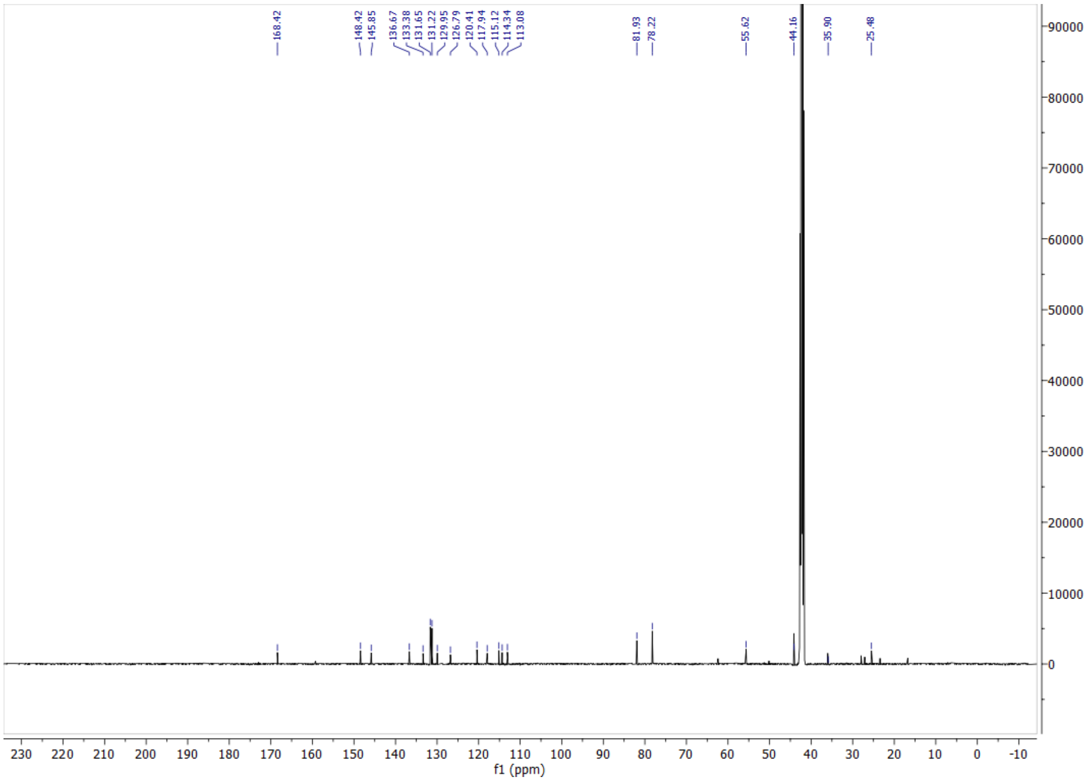

3-(2-(di(prop-2-yn-1-yl)amino)ethyl)-1*H*-indol-5-yl-(*E*)-3-(*p*-tolyl) acrylate (15)

^{1}H NMR (300 MHz, DMSO)

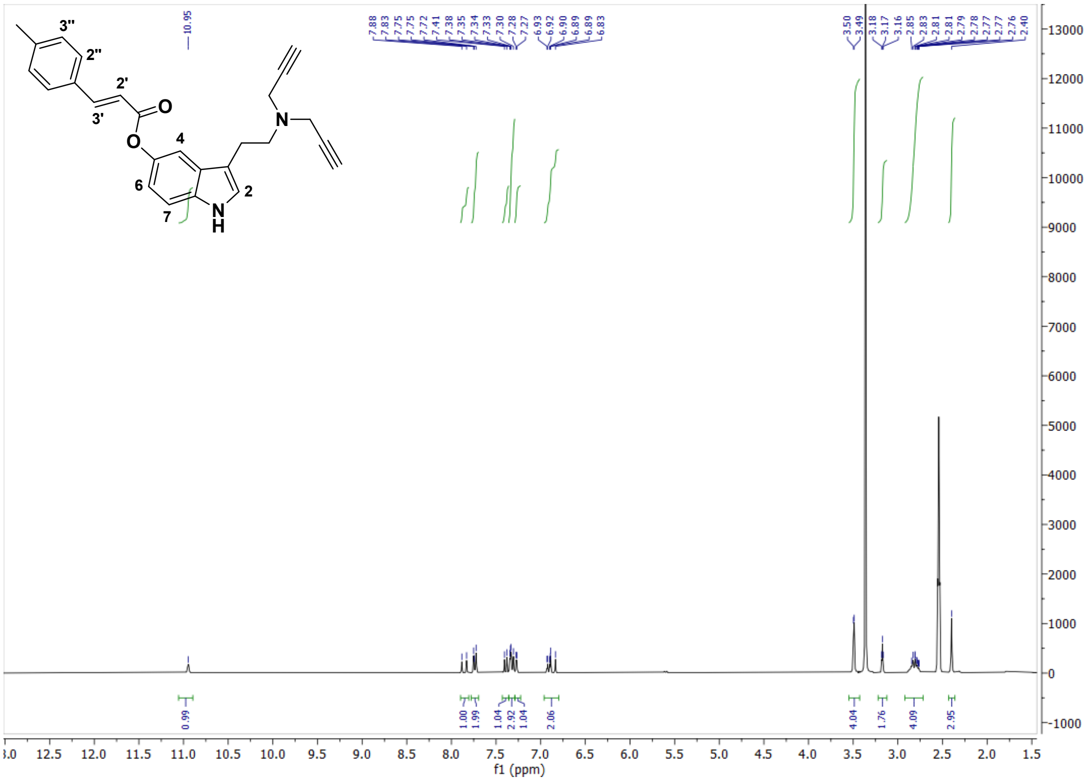

^{13}C NMR (75 MHz, DMSO)

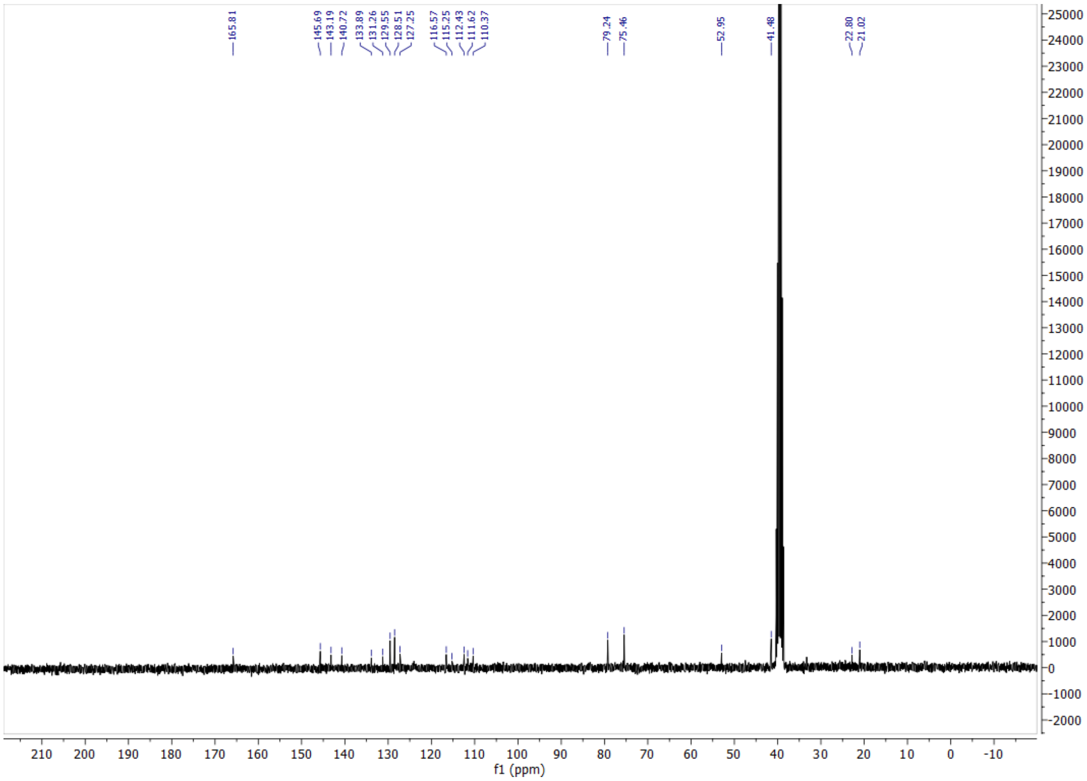

***N*-(2-(5-methoxy-1*H*-indol-3-yl)ethyl) propiolamide (16)**

^{1}H NMR (300 MHz, DMSO)

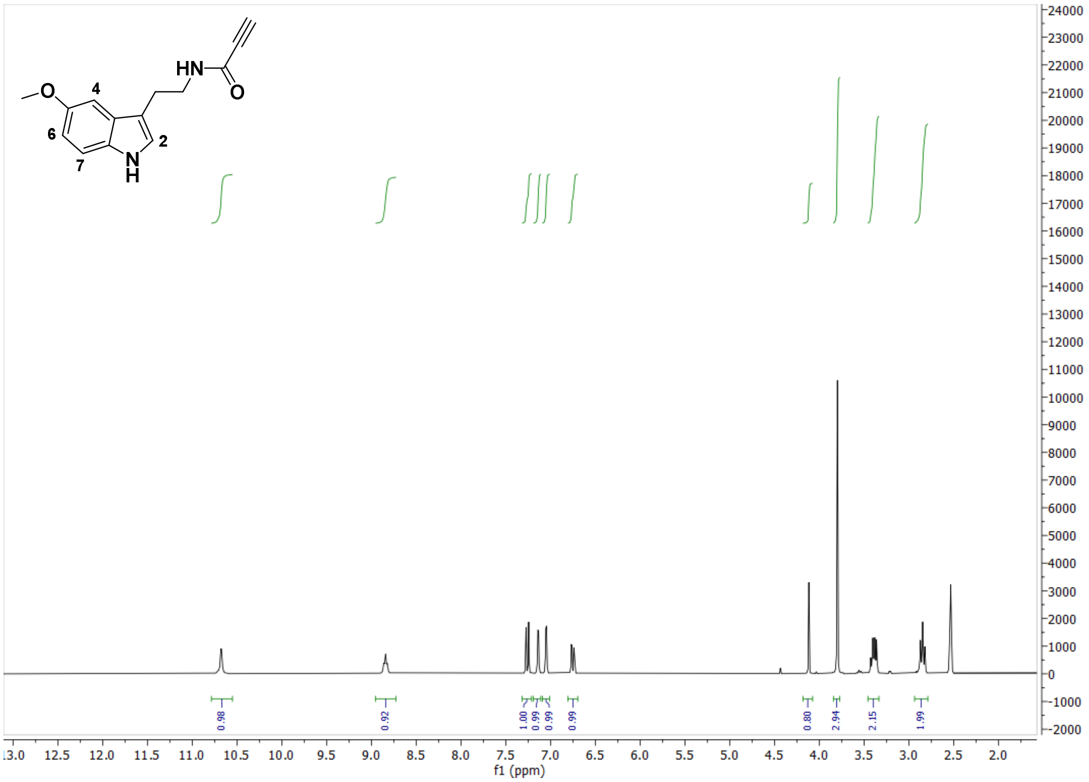

^{13}C NMR (75 MHz, DMSO)

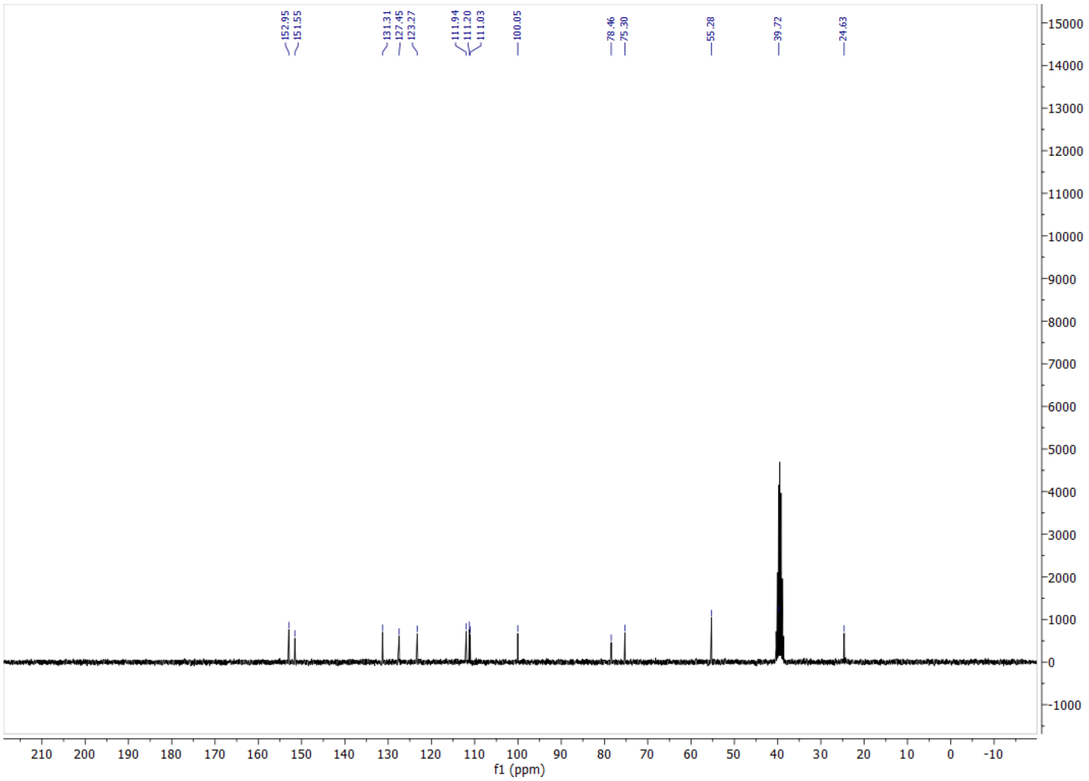

3-(2-propiolamidoethyl)-1*H*-indol-5-yl cinnamate (17)

^{1}H NMR (300 MHz, DMSO)

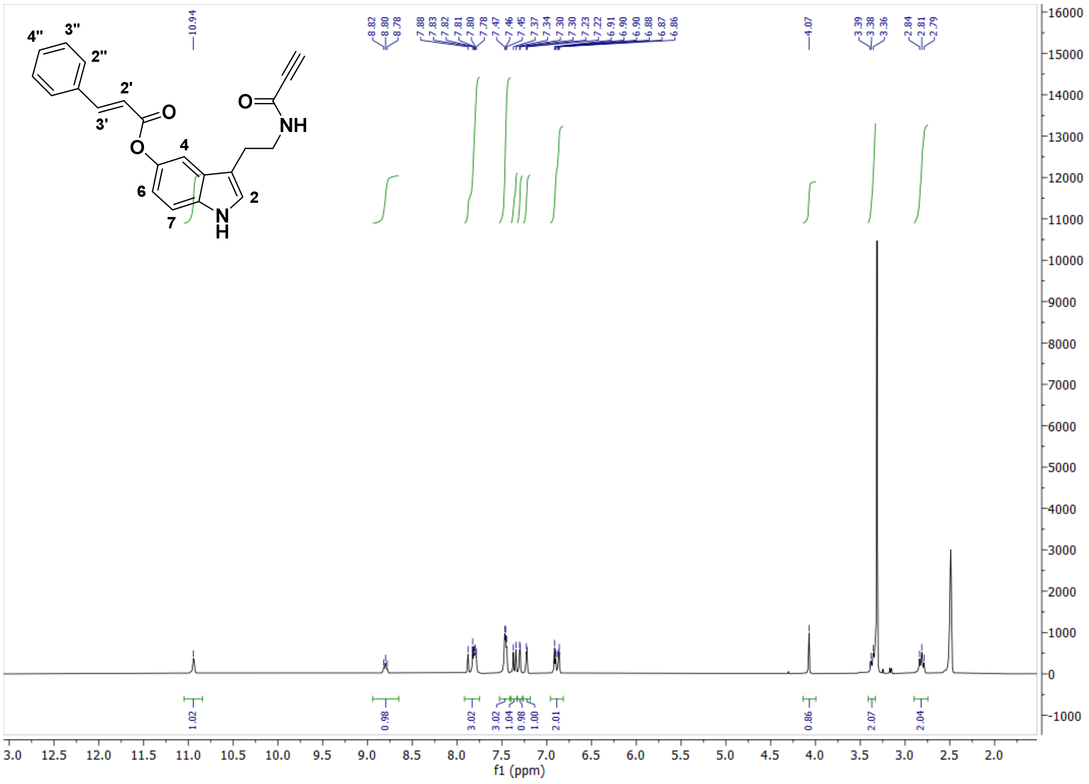

^{13}C NMR (75 MHz, DMSO)

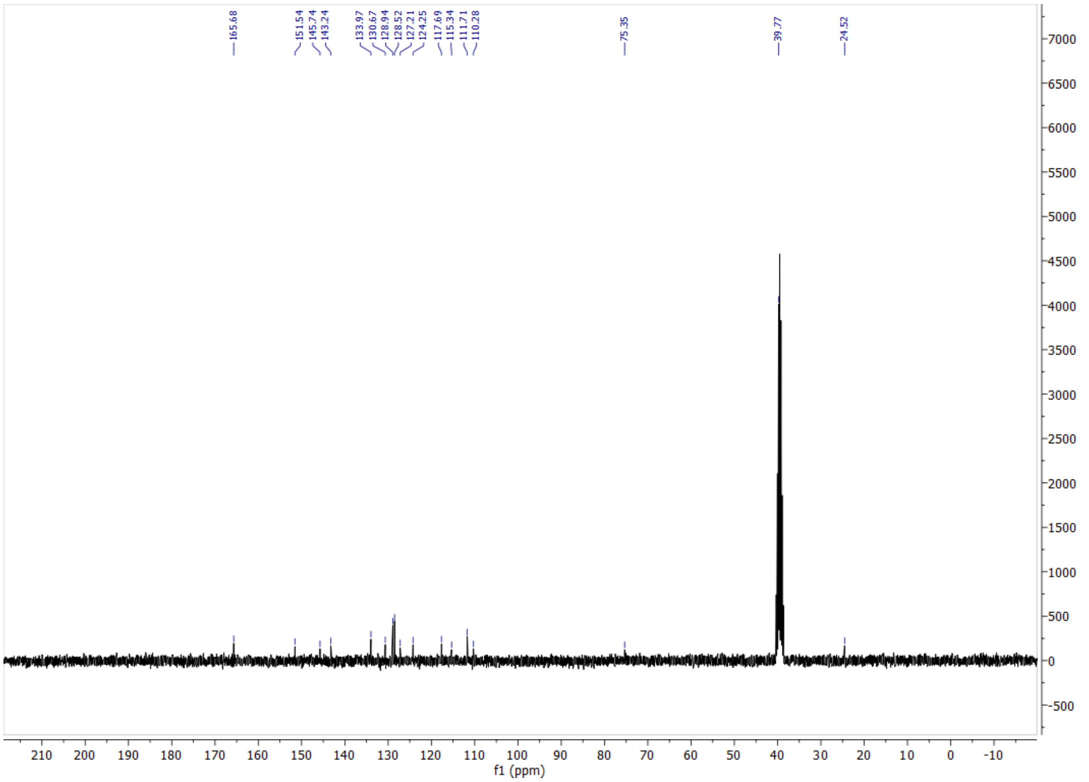

3-(2-propiolamidoethyl)-1*H*-indol-5-yl-(*E*)-3-(*p*-tolyl) acrylate (18)

^{1}H NMR (300 MHz, DMSO)

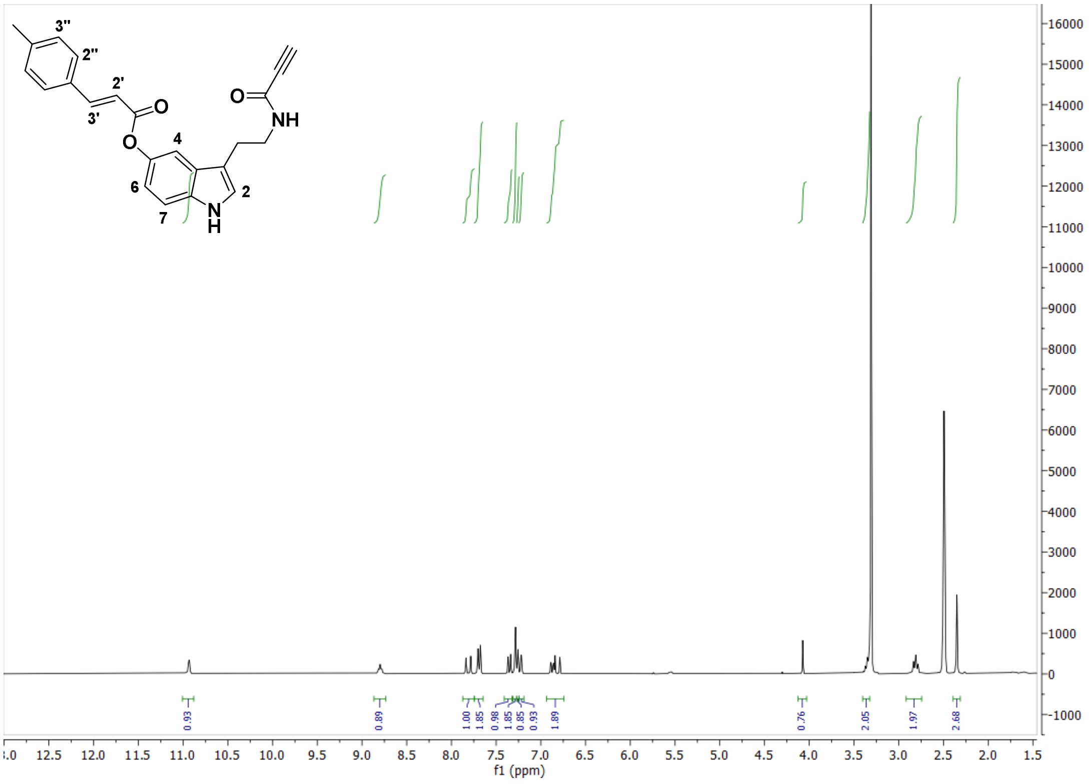

^{13}C NMR (75 MHz, DMSO)

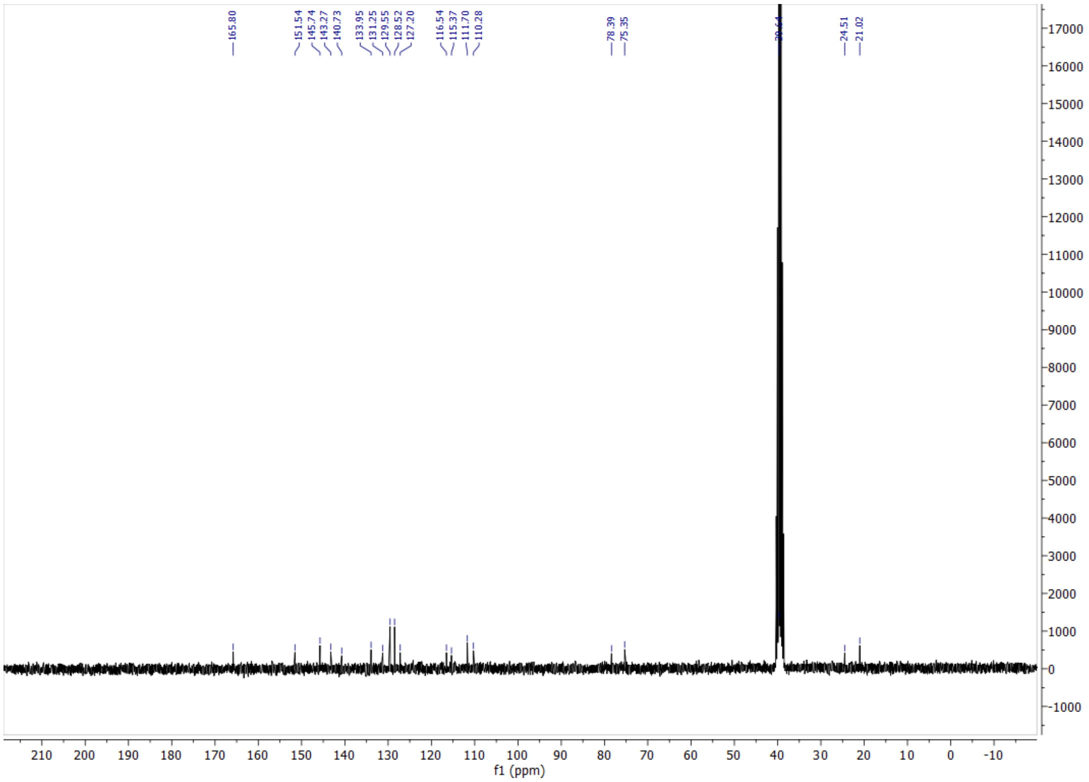

References

1. Daina, A., O. Michielin, and V. Zoete, *SwissADME: a free web tool to evaluate pharmacokinetics, drug-likeness and medicinal chemistry friendliness of small molecules.* Sci Rep, 2017. **7**: p. 42717.
2. Moriguchi, I., et al., *Simple method of calculating octanol/water partition coefficient.* Chemical and pharmaceutical bulletin, 1992. **40**(1): p. 127-130.
3. Moriguchi, I., et al., *Comparison of reliability of log P values for drugs calculated by several methods.* Chemical and pharmaceutical bulletin, 1994. **42**(4): p. 976-978.
4. Lipinski, C.A., et al., *Experimental and computational approaches to estimate solubility and permeability in drug discovery and development settings.* Adv Drug Deliv Rev, 2001. **46**(1-3): p. 3-26.
5. Ali, J., et al., *In Silico Prediction of Aqueous Solubility Using Simple QSPR Models: The Importance of Phenol and Phenol-like Moieties.* Journal of Chemical Information and Modeling, 2012. **52**(11): p. 2950-2957.
